# Cross-Species Annotation of Expressed Genes and Detection of Different Functional Gene Modules Between 10 Cold- and 10 Hot-Propertied Chinese Herbal Medicines

**DOI:** 10.3389/fgene.2020.00532

**Published:** 2020-06-18

**Authors:** Arong Li, Aqian Li, Zhijun Deng, Jiewen Guo, Hongkai Wu

**Affiliations:** ^1^Guangzhou Hospital of Traditional Chinese Medicine, Guangzhou University of Chinese Medicine, Guangzhou, China; ^2^Department of Pharmacy, Guangdong Hospital of Traditional Chinese Medicine, Guangzhou, China; ^3^Guangdong Key Laboratory of Mental Health and Cognitive Science, Center for Studies of Psychological Application, School of Psychology, South China Normal University, Guangzhou, China; ^4^State Key Laboratory of Respiratory Disease, National Clinical Research Center for Respiratory Disease, Guangzhou Institute of Respiratory Health, The First Affiliated Hospital of Guangzhou Medical University, Guangzhou Medical University, Guangzhou, China

**Keywords:** Chinese herbal medicine, property, transcriptome, RNA-seq, cross-species

## Abstract

According to the traditional Chinese medicine (TCM) system, Chinese herbal medicines (HMs) can be divided into four categories: hot, warm, cold, and cool. A cool nature usually is categorized as a cold nature, and a warm nature is classified as a hot nature. However, the detectable characteristics of the gene expression profile associated with the cold and hot properties have not been studied. To address this question, a strategy for the cross-species annotation of conserved genes was established in the present study by using transcriptome data of 20 HMs with cold and hot properties. Functional enrichment analysis was performed on group-specific expressed genes inferred from the functional genome of the reference species (i.e., *Arabidopsis*). Results showed that metabolic pathways relevant to chrysoeriol, luteolin, paniculatin, and wogonin were enriched for cold-specific genes, and pathways of inositol, heptadecane, lauric acid, octanoic acid, hexadecanoic acid, and pentadecanoic acid were enriched for hot-specific genes. Six functional modules were identified in the HMs with the cold property: nucleotide biosynthetic process, peptidy-L-cysteine *S*-palmitoylation, lipid modification, base-excision repair, dipeptide transport, and response to endoplasmic reticulum stress. For the hot HMs, another six functional modules were identified: embryonic meristem development, embryonic pattern specification, axis specification, regulation of RNA polymerase II transcriptional preinitiation complex assembly, mitochondrial RNA modification, and cell redox homeostasis. The research provided a new insight into HMs’ cold and hot properties from the perspective of the gene expression profile of plants.

## Introduction

In the system of traditional Chinese medicine (TCM), Yin–Yang theory is one of the central theories, which is used to explain how the world and body work. Yin and Yang represent the two ends of a spectrum like cold–hot, female–male, and inside–outside. When this concept is applied to the human body, Yin and Yang are linked to different parts or organs of the body or simply one’s feeling of cold and hot (Gu and Pei, 2017). Yin and Yang balance explains all changes and activities in nature, including the balance of life and body functions (Chan, 1995). The breaking of balance gives rise to different syndromes, which can be classified as “cold” syndromes and “hot” syndromes. These two types of syndromes therapeutically direct the use of Chinese herbal medicines (HMs) in TCM (Li et al., 2007). The “Yin–Yang” attribution is also used to define the nature of HMs, as HMs are generally featured as cold or hot. Actually, the nature of HMs consists of four types: hot, warm, cold, and cool, based on their interaction with human body. Cool nature usually is categorized as cold nature, and warm nature is classified as hot nature. For example, chewing a mint (*Mentha spicata*) leaf elicits a cold feeling, while masticating a piece of ginger (*Zingiber officinale*) root leads to a hot sensation. As a result, mint leaf is considered to be “cold” while ginger root is considered to be “hot” in nature (Zhao et al., 2011). Furthermore, in order to maintain a homeostasis in our body, the hot syndrome disease can be treated with cold nature HMs and cold syndrome disease with hot nature HMs (Chan, 1995). The cold and hot properties are the important medicinal properties of HMs in TCM theory.

The theory of four properties of HMs as an integral part of TCM is rooted from ancient Chinese philosophy. From the modern medical science point of view, it relies largely on the accumulation of experiences and the subjective opinions of TCM practitioners and therefore lacks objective, quantitative measurements and analysis. Since 1970s, with the introduction of modern science and technology, research on the properties of HMs have made some progress, and new ideas and methods have been appeared constantly, including micro-calorimetry (Ren et al., 2009; Jia et al., 2010; Zhao et al., 2011), support vector machine (SVM) (Ung et al., 2007), biophoton radiation detection (Zhao and Han, 2013), predicting system based on chemical material basis (Long et al., 2011), etc. According to different modern scientific instruments and methods, the properties of HMs were summarized by researchers from different perspectives. For example, from the thermokinetic point of view, the herbs that caused the body to release heat owned the “hot” property, and those that absorbed the heat owned “cold” property (Zhao et al., 2011). Some pharmacological studies supported the notion that the cold and hot properties of HMs were closely related to excitability of nervous system and endocrine (Li et al., 2008; Fu et al., 2009), mitochondrial ATP generation, and immunomodulatory function (Ko et al., 2004, 2006; Ko and Leung, 2007). Moreover, research on the functions of HMs’ targeted proteins suggested that hot propertied HMs were more related to inflammation and immunity regulation and that cold-propertied HMs possessed tendencies in cell proliferation (Liang et al., 2013). From a chemical point of view, the herbal compounds associated with cold nature generally possessed more polar structures. Their molecular weights were lower, in contrast to the compounds associated with hot nature (Fu et al., 2017; Huang et al., 2018). This research shows that there are evident differences between cold and hot HMs in the chemical composition or the efficacy and cognitive experience gained from patient response. To date, there is no report on the research of the cold and hot properties of HMs from the perspective of plant gene expression profile based on RNA-seq.

With the advent of high-throughput RNA-seq, there has been a concerted effort on generating a whole transcriptome of plant species (Wang et al., 2009; Zeng et al., 2010; Wenping et al., 2011; Yuan et al., 2012; He et al., 2013). Transcriptome studies have focused on functional genes and metabolic pathways and provided molecular basis for metabolic pathways of natural drugs (Soetaert et al., 2013; Gao et al., 2014). As previous studies mainly focused on one species, integrating data of multiple species in a reasonable way will be more informative to study the gene function relationship between different properties of HMs. Indeed, cross-species meta-analysis of gene expression profiles has previously been used to address many questions in plants, such as the adaptation to progressive drought stress (Shaar-Moshe et al., 2015). In the present study, a method for cross-species comparison of plant was established to investigate the differences between cold and hot properties of HMs in the active gene level.

Until now, few computational methods have been proposed and developed to analyze interspecies gene expression data. Fisher’s combined probability test, which transforms *p*-values from any number of tests into one single *p*-value, has been a popular method for comparing multiple gene expression experiments (Campain and Yang, 2010; Tseng et al., 2012; Matur and Ulgen, 2019). An approach was developed to compare gene expression of homologs (identified using reciprocal best BLAST hits) over a wide range of experimental conditions (Stuart et al., 2003). Another method called mDEDS combined several different statistical measures to perform a cross-species comparison of gene expression profiles (Le et al., 2010). Other methods include LOLA (Cahan et al., 2005) and L2L (Newman and Weiner, 2005), which are both online tools for comparisons among different species by ranking lists of differentially expressed genes from microarray studies. These methods were applied to comparisons among species that are related relatively closely in phylogenetic terms, such as mice and humans. Furthermore, a method for comparing gene expression among distant species was developed by taking the homology structure of compared species into account and comparing expression data from genes with any number of orthologs and paralogs (Kristiansson et al., 2013). However, the homology mapping mainly depended on whether these species had available well-annotated genomes (e.g., model species). As HM many plants are less common (non-model species) and distantly related species, such as gymnosperm *Ephedra sinica* and angiosperm *Scutellaria baicalensis*, these methods did not seem to be suitable for most species in this study. Therefore, a method for cross-species comparison of less common and distant species was needed to be established.

Benchmarking Universal Single-Copy Ortholog system (BUSCO^1^) (Waterhouse et al., 2018) proposed a measure for the assessment of assembled transcriptomes with single-copy orthologs. Specifically, BUSCO assessment tool used the orthologs of 31 plants from OrthoDB^2^ (Zdobnov et al., 2017) to produce 1,440 BUSCO gene sets from plant phylogenetic clades. By using HMMER 3 (Eddy, 2011), hidden Markov model (HMM) profiles were obtained from amino acid alignments built with these 1,440 gene sets. Then, the transcriptome completeness information (C: single and completed; D: duplicated and completed; F: fragmented; M: missing) of plant phylogenetic clades could be assessed, which provided a way to find out the orthologs (C: single and completed) of less common and distant plant species. We could use these orthologs (C: single and completed) to establish our cross-species comparison standard. That is, with the aid of BUSCO, ortholog and phylogenetic information could be taken into account during the gene annotation process. The flowering plant *Arabidopsis thaliana* is a dicot model organism for research in many aspects of plant biology. The comprehensive annotation of its genome paves the way for understanding the functions and activities of all types of transcripts, including mRNA, the various classes of non-coding RNA, and small RNA (Provart et al., 2016). The maintained database TAIR11 completely recorded its genetic and molecular biology data (Cheng et al., 2017). Therefore, the main annotation strategy in this study was to use the model organism *A. thaliana* as the reference to compare any other query species to annotate genes.

In this study, 20 sets of available plant RNA-seq data (HMs with the cold and hot properties) from 20 published papers were collected. We first created a comparison standard for cross-species designation of gene function based on BUSCO results, applied it in BLASTP search, and then annotated their genes. With the specifically expressed gene on hot or cold HMs, we finally performed functional enrichment analysis to identify differences between hot-enriched and cold-enriched functional modules. From the perspective of gene expression profiles, the results might provide useful new clues for exploring measurable features of cold and hot HMs with their original definition.

## Materials and Methods

### Selection of HMs With Cold and Hot Properties

To obtain sufficient data for HMs, we reviewed the advances in HMs transcriptome studies. Specifically, the databases MEDLINE^3^, Embase^4^, and Google Scholar^5^ were searched for articles published up to January 01, 2018. For the information associated with HMs, such as TCM properties, medicinal organs of plants were searched from *Chinese Medicine Dictionary* (Shanghai Science and Technology Press, Second Edition) and listed as basic information. The collected data were filtered, and HMs datasets only from Illumina paired-end sequencing with similar read lengths were kept for further study. In order to achieve even organ distribution between the two categories of HMs, 5 samples for roots, stems, leaves, and flowers were selected, respectively. In this way, a total of 10 cold and 10 hot HMs were enrolled (Table 1). The phylogenetic tree of species was constructed by NCBI Taxonomy tools^6^.

**TABLE 1:** Selected Chinese herbal medicines.

Property	Sample id	Species	Organs	HM Chinese name	HM Latin name	SRR id	References
Cold	cold1	*Rehmannia glutinosa* (Gaertn.) Libosch.	Roots	Xian Di Huang	*Radix Rehmanniae recens*	SRR832972	Li et al., 2013
	cold2	*Dracocephalum tanguticum* Maxim.	Leaves	Tang Gu Te Qing Lan	*Herba Dracocephali Tangutici*	SRR2915458	Li et al., 2017
	cold3	*Scutellaria baicalensis* Georgi	Roots	Huang Qin	*Radix Scutellariae*	SRR3367956	Liu J. et al., 2015
	cold4	*Catharanthus roseus* (L.) G. Don	Flowers	Chang Chun Hua	*Herba Catharanthi Rosei*	SRR1271859	Verma et al., 2014
	cold5	*Andrographis paniculata* (Burm. f.) Nees	Leaves	Chuan Xin Lian	*Herba Andrographis*	SRR1519324	Garg et al., 2015
	cold6	*Swertia mussotii* Franch.	Flowers	Zang Yin Chen	*Herba Swertiae Mussotii*	SRR3951703	Liu et al., 2017
	cold7	*Gentiana rigescens* Franch. ex Hemsl.	Roots	Long Dan	*Radix Gentianae*	SRR924095	Zhang X. et al., 2015
	cold8	*Gardenia jasminoides* Ellis	Petals	Zhi Zi Hua	*Flos Gardeniae Jasminoidis*	SRR1045129	Tsanakas et al., 2014
	cold9	*Lonicera japonica* Thunb.	Flowers	Jin Yin Hua	*Flos Lonicerae*	SRR3591711	Rai et al., 2017
	cold10	*Isatis tinctoria* Fort.	Leaves	Qing Dai	*Indigo Naturalis*	SRR1565773	Zhou et al., 2015
Hot	hot1	*Allium fistulosum* L.	Leaves	Cong Ye	*Folium Allii Fistulosi*	SRR1609976	Liu et al., 2014
	hot2	*Isodon rubescens* (Hemsl.) Hara	Leaves	Dong Ling Cao	*Herba Rabdosiae Rubescentis*	SRR5367856	Su et al., 2016
	hot3	*Curcuma longa* L.	Rhizomes	Jiang Huang	*Rhizoma Curcumae Longae*	SRR3928562	Sahoo et al., 2016
	hot4	*Atractylodes lancea* (Thunb.) DC.	Rhizomes	Cang Zhu	*Rhizoma Atractylodis*	SRR3104394	Huang et al., 2016
	hot5	*Erigeron breviscapus* (Vant.) Hand. -Mazz.	Flowers	Deng Zhan Xi Xin	*Herba Erigerontis*	SRR1867750	Zhang W. et al., 2015
	hot6	*Ephedra sinica* Stapf	Shoot tip	Ma Huang	*Herba Ephedrae*	SRR1188607	Groves et al., 2015
	hot7	*Anemone flaccida* Fr. Shmidt	Stems	Di Wu	*Rhizoma Anemones Flaccidae*	SRR3233423	Zhan et al., 2016
	hot8	*Pinellia ternata* (Thunb.) Berit	Tubers	Ban Xia	*Rhizoma Pinelliae*	SRR1186931	Zhang G. et al., 2016
	hot9	*Panax notoginseng* (Burk.) F. H. Chen ex C. Chow	Roots	San Qi	*Radix Notoginseng*	SRR1032053	Liu M. et al., 2015
	hot10	*Lindera glauca* (Sieb. et Zucc.) Bl	Roots	Shan Hu Jiao Gen	*Radix Linderae*	SRR1438496	Niu et al., 2015

### *De novo* Assembly and Assessment of the Transcriptome Assemblies

The RNA-seq data sets were downloaded from the NCBI Sequence Read Archive (SRA^7^) according to their SRR IDs recorded in 20 articles (Table 1). Then, all reads were processed through a trimming pipeline using Trimmomatic (version 0.32, default parameters) (Bolger et al., 2014) to remove residual adapters, low-quality sequences, and reads below 36 bp. FastQC^8^ was used an overview for sequencing quality. The remaining high-quality reads were *de novo*-assembled into candidate unigenes longer than 200 bp by using Trinity (version 2.6.6, default parameters) (Grabherr et al., 2011). The abundance of each transcript and gene measured by the value of transcripts per million (TPM) was calculated by using the script *align_and_estimate_abundance.pl* from Trinity software package. Here, RSEM (Li and Dewey, 2011) was called to compute TPM after alignments were done by Bowtie2 (Langmead and Salzberg, 2012).

TransRate (Smith-Unna et al., 2016) was used to evaluate the transcriptome assembly contiguity by producing a score based on length-based and mapping metrics. BUSCO (version 3.0), which evaluates assembly content by searching the assemblies against conserved single copy orthologs found in all Embryophyta, was applied for further quantitative assessment of the assembly completeness.

### Cross-Species Gene Annotation

**FIGURE 1:**
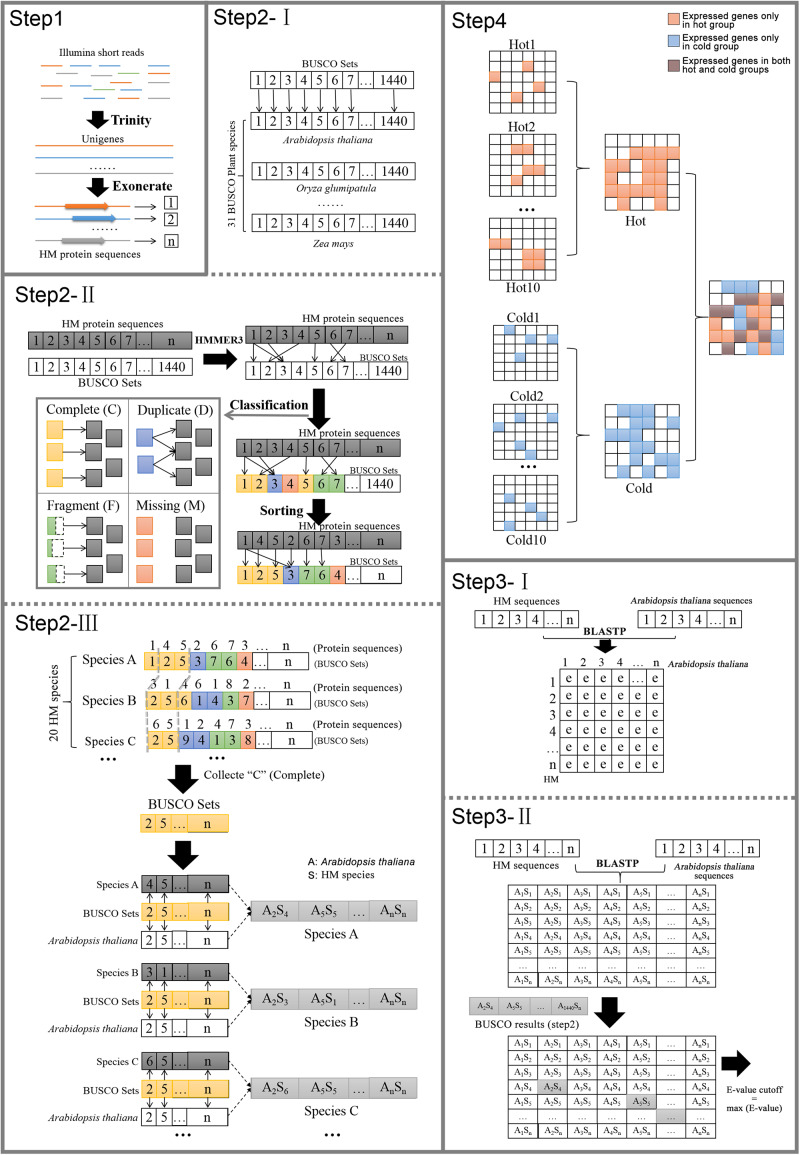
Pipeline of cross-species gene annotation and selection of specific expressed genes.

*Arabidopsis thaliana* protein sequences and annotation information downloaded from TAIR11 were selected as reference. The distances of phylogenetic relationships between *A. thaliana* and each species in our research varied greatly; some species such as gymnosperm *E. sinica* and angiosperm *S. baicalensis* were distantly related. The annotations of genes were assigned by the reference genes through the searching results of BLASTP (Camacho et al., 2009). Among the species, the results would be greatly influenced by the evolutionary distance from reference if uniform cutoff was set, because, at the same sensitivity, the longer distance the species to which the sequence belonged, the less possibility it was to hit the homologous gene. So, it was necessary to establish the reasonable cutoff value for each species separately by considering its evolutionary distance. Meanwhile, the *E*-value, which considered the size of the database and the scoring system, provided an indication of the statistical significance of a given pairwise alignment. Therefore, *E*-value was chosen as the cutoff, and it must be that when the phylogenetic relationships between the species and the reference are farther, the looser they are. The process was as follows (Figure 1).

Step 1: Identify species candidate protein sequences. For each species, the transcriptome was assembled *de novo* from the RNA-seq reads by using Trinity. The sequences were trimmed down to open reading frame by using Exonerate (Slater and Birney, 2005). The longest transcript was extracted for each protein-coding gene locus after confirming the presence of start and stop codons and the proper reading frame. The coding sequences were then translated into protein sequences.
Step 2: Identify orthologs of HM plants conserved genes in *Arabidopsis*. This step was to classify the candidate conserved genes form 20 plants into BUSCO orthologs and establish gene pairs of HM plants and Arabidopsis.
  (I) OrthoDB^2^ was a comprehensive catalog of orthologs. In 2016 OrthoDB reached its 9th release, which contained more than 22 million genes from over 5,000 species. BUSCO used OrthoDB to define 1,440 BUSCO sets, which contained gene orthologs from 31 species of plants (including *A. thaliana*).
  (II) The HMMER3 HMM profiles from amino acid alignments were used to assess whether a protein sequence from species could join the orthologs of distinct BUSCO set and to classify the matches into four categories of C, D, F, or M.
  (III) HMMER3 matching was performed on each species separately, and only the newly formed orthologs of C were retained for subsequent steps. Among genes in the same orthologs, the gene from HM species and the gene from *A. thaliana* were picked up to form a gene pair.
Step 3: Define BLASTP *E*-value cutoff values for each species.
  (I) In each HM species, BLASTP was performed with default parameter between the protein sequences of *A. thaliana* and the candidate protein sequences of each species to get the hits and their similarity information including *E*-value.
  (II) In each HM species, among the matched gene pair identified by BLASTP, those corresponding to the gene pairs obtained by using BUSCO (Step 2) were found. Their *E*-values were collected for 20 species, respectively, and the maximum value of individual species was taken as the *E*-value cutoff of the species.
Step 4: Another round of BLASTP was run for each HM species by setting the species-specific *E*-value cutoff yielded from Step 3, again using *A. thaliana* as reference. The gene function information was finally assigned according to its matched gene of *A. thaliana*. It should be noted that one reference sequence might be matched by more than one HM sequence, so there are cases where multiple genes in same species shared the same annotation. Meanwhile, the genes that were expressed only in cold HMs or only in hot HMs were figured out, as well as genes that were expressed only in specific organ, dicotyledons or non-dicotyledons. The reference genes that matched their counterparts only in a certain group of HMs were defined as group-specific genes.

To further validate the reliability of the newly formed orthologs and the cross-species annotation of 20 HMs conserved genes, we conducted two types of analysis accordingly:

(1) Another model organism (*Oryza sativa Japonica Group*) was selected as the outgroup. In each newly formed ortholog (Step 2), the conserved genes of 20 HMs and the outgroup were collected. These multi-sequence data sets were, respectively, aligned by using MAFFT (v7.305, options –thread - 12 –auto) (Katoh and Standley, 2013) and filtered by using trimAl (v1.4 rev10, option -automated1) (Capella-Gutierrez et al., 2009). Then, all the alignments were concatenated, and the maximum likelihood tree was produced by using RAxML (v8.1.2, raxmlHPC-PTHREADS-SSE3 -T 12 -f a -m PROTGAMMAJTT -N 100 -n rodents -s $wd/supermatrix.aln.faa -p 13432 -x 89090) (Stamatakis, 2014). The resulting tree was rooted by newick utilities (v1.6) (Junier and Zdobnov, 2010). The overall validity of conservation for the annotated genes from 20 HMs could be verified by comparing the inferred phylogenetic relationships with the NCBI Taxonomy tree.
(2) Model organism (*O. sativa Japonica Group*) was selected as another reference by using database Ensembl. The gene function information of 20 HMs’ conserved genes was assigned according to its best matched gene of *O. sativa* by using BLASTP. The reliability of cross-species annotation of 20 HMs’ conserved genes was evaluated by comparing consistency of annotation results between using *A. thaliana* as the reference and using *O. sativa* as the reference. On the one hand, the proportions of the genes with consistent annotations were manually inspected and counted. On the other hand, genes of *A. thaliana* matched to HMs were assigned to *Oryza sativa* using BLASTP. The pairs of genes from *A. thaliana* and *O. sativa* were obtained. Then, the proportions of the pairs of which both genes were matched the same HM sequence were calculated.

### Gene Function Module Enrichment Analysis

Functional enrichment analysis was performed on group-specific genes by using Metascape^9^, an online bioinformatics pipeline for multiple gene lists, which supports effective data-driven gene prioritization decisions (Zhou et al., 2019). The analysis workflow included (i) ID conversion of input gene identifiers into Entrez gene IDs of *A. thaliana*; (ii) extraction of annotations for the gene list using GO ids and KEGG Pathways ids; and (iii) functional enrichment analysis through the gene list. Functional categories of GO Molecular Functions, GO Cellular Components, GO Biological Processes GO were applied for the analysis of organ-specific and dicotyledon-/non-dicotyledon-specific genes, and GO Biological Processes and KEGG Pathways were applied for cold/hot specific genes. All genes in the reference genome (*A. thaliana*) were used as the enrichment background and the filtering criteria for the results were, minimal number of overlap genes ≥ 3, enrichment factor > 1.5, and *P*-value < 0.01. Remaining significant terms were then hierarchically clustered into a tree based on Kappa-statistical similarities among their gene memberships. A kappa score of 0.3 was applied as the threshold to cast the tree into term clusters. A subset of up to 10 representative terms from each of the 20 top-score clusters were then selected and converted into a network layout. Networks were visualized through Cytoscape (v3.1.2) (Bauer-Mehren, 2013) using ‘force-directed’ layout with edges bundled for clarity. For identifying enrichment of specific terms of interest, a term with FDR < 0.05 was considered significant.

### Statistical Analysis for Between-Group Comparisons

The Illumina sequencing and transcriptome assembly results were compared between cold and hot properties of HMs. For normally distributed data, the homogeneity of variance test was conducted first. *T*-test was performed for those with homogeneity of variance, and *t*′-test for those with missing variance. For non-normally distributed data, Mann–Whitney *U* non-parametric test was performed. All tests were performed using a two-tail hypothesis, with significance set at *P* < 0.05.

### Acquisition of Chemical Components of HMs and Their Metabolic Pathways

All of the chemical ingredients of HMs were collected from BATMAN-TCM (Bioinformatics Analysis Tool for Molecular mechANism of TCM) (Liu Z. et al., 2016), an online bioinformatics analysis tool for studying the molecular mechanism of TCM. As components of cold2 (*Herba Dracocephali Tangutici*), cold6 (*Herba Swertiae Mussotii*), hot7 (*Rhizoma Anemones Flaccidae*) and hot10 (*Radix Linderae*) were not recorded in the database, chemical ingredients for these four HMs were obtained from literature (Zhang et al., 2008; Zuo et al., 2015; Chen et al., 2016; Liu T. et al., 2016; Yang et al., 2016; Wang H. et al., 2017; Xiang et al., 2019; Yixi et al., 2020). For the cold or hot group, the components that were shared by at least two HMs in one group and were not contained in the other group were regarded as the group-specific components. The metabolic pathways of group-specific chemical ingredients were searched through KEGG database^10^.

## Results

### Statistical Results of HMs High-Throughput Transcriptome Literature Data

**FIGURE 2:**
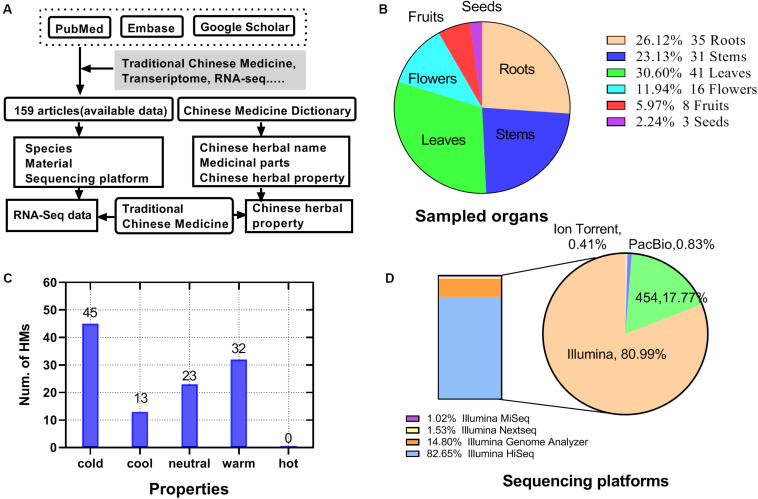
Statistics for HMs high-throughput transcriptome literature data. **(A)** Source and process of data acquisition of high-throughput transcriptome and HMs’ information. **(B)** Distribution of sampled organ of HMs. **(C)** Quantity of HMs for each property. **(D)** Distribution of types of sequencing platforms.

Up to January 01, 2018, 159 articles related to HM and transcriptome were identified (Supplementary Table S1). The information collection process was shown in Figure 2A. Among the articles, 113 HMs were screened, including 32 with the hot property (32 with warm property and 0 with hot property), 58 with the cold property (45 with cold property and 13 with cool property), and 23 with a neutral property (Figure 2C). Of the sampled organs from HMs, the largest proportion was 30.60% in leaves, followed by roots, stems, flowers, and fruits, accounting for 26.12, 23.13, 11.94, and 5.97%, respectively. The proportion of seeds was the smallest, only 2.24% (Figure 2B). There were four types of platforms used for sequencing reported in the articles, namely Ion Torrent, PacBio, 454, and Illumina (Figure 2D). Among them, Illumina covered 81% of the total, which came to be the most commonly used sequencing platform for HM transcriptome sequencing, followed by 454 (18%). Illumina’s models included Illumina miseq (1.02%), Illumina nextseq (1.53%), Illumina genome analyzer (14.80%), and Illumina hiseq (82.65%).

### Selection of HMs With Cold and Hot Properties and *de novo* Assembly

**FIGURE 3:**
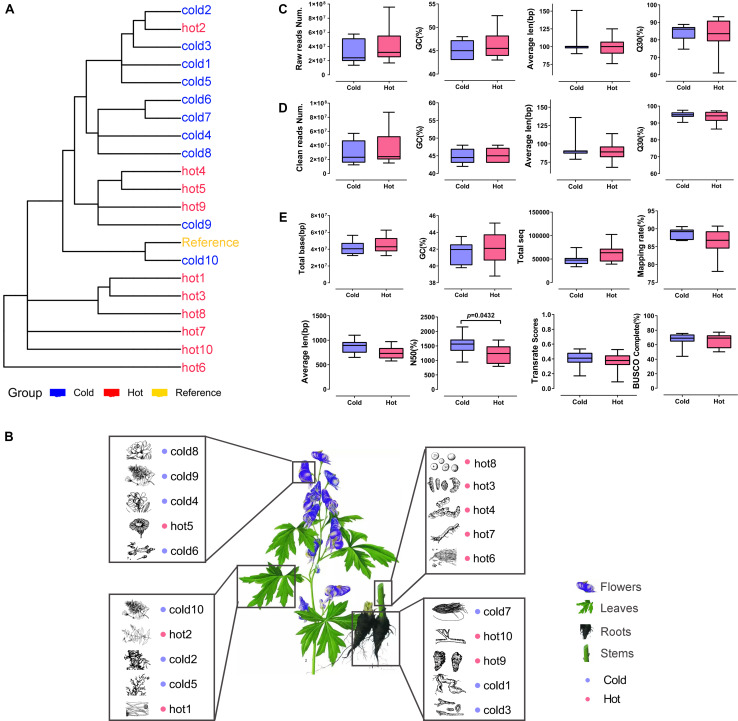
Phylogenetic relationship, organ distribution, qualities of sequencings, and assemblies of 20 samples. Red color indicates HMs with the hot property; blue color indicates HMs with the cold property. **(A)** Phylogenetic relationship of species used in the study. **(B)** Organs of selected HMs. The pictures of HMs were cited from *Chinese Medicine Dictionary* (Shanghai Science and Technology Press, Second Edition). **(C,D)** Sequencing qualities before and after quality control. **(E)** Statistics for *de novo* transcriptome assembly after quality control.

As shown in Table 1, 10 cold and 10 hot propertied HMs were recruited into the analysis. The selected samples were evenly distributed among organs (Figure 3B). The phylogenetic tree of our selected 20 species showed that the closest species to the reference specie (*A. thaliana*) was *Isatis tinctoria* (cold10), and the farthest was *E. sinica* (hot6) (Figure 3A). The species in this lineage contained dicotyledonous and non-dicotyledonous plants and were from Magnoliophyta, except for hot6 from Gnetophyta (Supplementary Table S2).

The RNA-seq datasets were downloaded according to their SRR IDs (Table 1). After the removal of adaptor, ambiguous reads, and low-quality reads, the GC content was 42–48%, the percentage of Q20 of each sample was 100%, and the percentage of Q30 was 86.43–97.56% (Supplementary Table S3). The FastQC results of each sample before and after quality control were shown on Supplementary Figure S1. All the clean reads from each sample were brought together and assembled *de novo* by using Trinity, respectively, generating 33,605 ∼ 102,400 unigenes. The average lengths of the unigenes were 577 ∼ 1,101 bp, and the lengths of N50 were 796 ∼ 2,155 bp. The length distribution of unigenes was shown in Supplementary Figure S2. The TransRate assembly score was 0.09014 ∼ 0.53457 and the BUSCO completeness range was 44 ∼ 77.3% (Supplementary Table S4).

The Illumina sequencing and transcriptome assembly results were compared between cold and hot HMs, including reads number, GC content, average length, base sequencing accuracy, total number of reads, total number of bases, mapping rate, BUSCO completeness, TransRate assembly score, and N50. The results showed that except for N50 (*P* = 0.0432), all the other variables listed above revealed no significant differences between cold and hot groups (*p*-values > 0.05, Figures 3C–E).

### Cross-Species Gene Annotation Results

The abundances of unigenes were comprehensively surveyed. In most species, the number of genes with TPM < 1 [log10(TPM) < 0] accounts for a very small proportion, and the cumulative frequency of genes increases rapidly after TPM < 1 (Supplementary Figure S3). As the computing of TPM had been weighted by library size, and in order to choose genes only with strong expression evidences, TCM > 1 was set as the filtering criterion to collect candidate genes for analyses.

**FIGURE 4:**
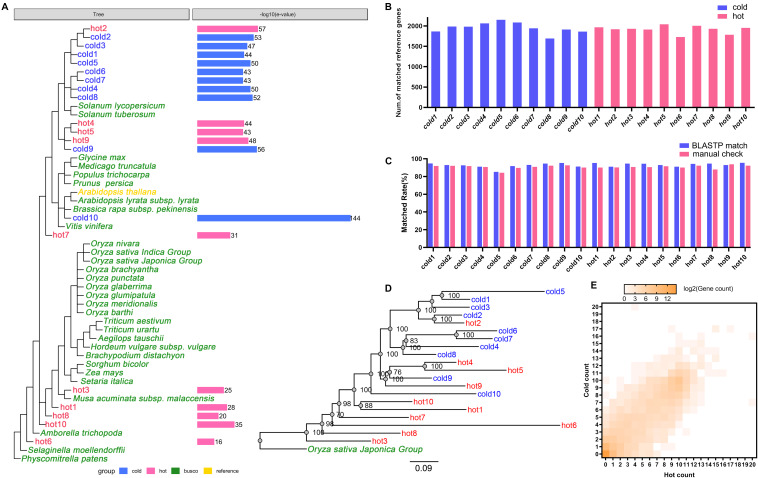
Cross-species annotation of expressed genes in 20 species. Red color indicates HMs with the hot property, blue color indicates HMs with the cold property, yellow color indicates reference species, and green color indicates 31 plants from 1,440 BUSCO sets. **(A)** *E*-value cutoff parameters of 20 samples in BLASTP. The phylogenetic tree showed the relationships of 20 HMs samples, the reference, and 31 plants from BUSCO. The column graphs on the right side showed the *E*-value cutoff which had been converted to –log10(*E*-value). **(B)** Read alignment counts of 20 samples. The difference of alignment counts was not statistically significant (*P* > 0.05). **(C)** Coincidence rates of the annotation results between using *Arabidopsis thaliana* as the reference and using *Oryza sativa* as the reference. Blue color indicates the coincidence rates were obtained by using BLASTP match; red color indicates the coincidence rates were obtained by manual comparison. **(D)** The maximum likelihood phylogenetic tree of 20 HMs and *O. sativa* constructed by concatenating the sequences of conserved genes. The numbers on the branches were the consistent quantities derived from bootstrap analysis. **(E)** The heat map of reference gene numbers according to the numbers of species genes (axis *X*-/*Y*-) in each group which matched to the reference genes. The color key from gray to brown indicates small to large matched gene numbers.

Among expressed genes, some conserved genes were classified into BUSCO sets (Supplementary Data S1/BUSCO result). With these genes in all 20 species, new complete single-copy orthologs were found. Among the gene items of the orthologs, the gene pairs of genes from sample species and a gene from *A. thaliana* were identified (Figure 1, Step 2-III). Similarity indicators for these gene pairs obtained by BLASTP through the default parameter (Supplementary Data S1/BLASTP default parameter) were identified. For each species, maximum *E*-value on this list was taken as cutoff for corresponding species and applied to the final annotation. Figure 4A shows the phylogenetic tree of 20 samples, 31 species from BUSCO, and each sample’s *E*-value cutoff, which was converted to “−log10(*E*-value).” *Isatis indigotica* (cold10) and *A. thaliana* were closest in genetic relationship, as they both belonged to Cruciferae. The value of *I. indigotica* was 114, which was the largest, while *Gymnosperms ephedra* (hot6) was the farthest species from *A. thaliana*, with a value of 16, the smallest. The values of *Isodon rubescens* (hot2), *Dracocephalum tanguticum* (cold2), *S. baicalensis* (cold3), *Rehmannia glutinosa* (cold1), *Andrographis paniculata* (cold5), *Swertia mussoi* (cold6), *Gentiana rigescens* (cold7), *Catharanthus roseus* (cold4), *Gardenia jasminoides* (cold8), *Atractylodes lancea* (hot4),*Erigeron breviscapus* (hot5), *Panax notoginseng* (hot9), and *Lonicera japonica* (cold9), which belongs to Compositae, ranged from 43 to 57. The values of *Curcuma longa* (hot3), *Allium fistulosum* (hot1), *Pinellia ternata* (hot8), and *Lindera glauca* (hot10), which belonged to Monocotyledonous, far away from *A. thaliana*, were lower than 35. These results show the tendency that the larger the evolutionary distance between a sample and *A. thaliana*, the greater the *E*-value, which made the next round of BLASP for gene annotation more sensitive in species that were distantly related to the reference. By applying the specific *E*-value as BLASTP cutoff for each species (Supplementary Data S1/BLASTP specific cutoff), there were 1,693–2,152 distinct reference genes matched by genes of 20 samples (Figure 4B), and no significant difference in quantity between hot and cold groups (*P* = 0.5787 > 0.05). The assembled sequences and their annotations are available by request through email. There were 27,416 proteins of *A. thaliana* collected in TAIR11; among them, 4,810 (17.54%) in total were hit by HM proteins. In each group (cold/hot) of HMs, the numbers of proteins that hit each single protein of the reference were counted. The numbers of reference genes were counted according to how many proteins were hit (Figure 4E). The heat map showed that the numbers of reference genes which matched different numbers of the genes of cold or hot propertied HMs were generally evenly distributed on both sides of the diagonal, indicating that there was no obvious cold or hot bias under the annotation process.

Besides, the inferred phylogenetic tree (Figure 4D) through concatenated conserved gene sequences rooted with *O. sativa* showed good agreement with the species phylogeny (Figure 4A). There was minor deviation on hot1 and hot10, which might be due to the distant evolutionary distance relative to other plants. The coincidence rates of the annotation results between using *A. thaliana* as the reference and using *O. sativa* as the reference were 85.32–95.39% or 84.20–93.77% (by the BLASTP match or the manual check, Figure 4C). These results showed that the cross-species annotation of conserved genes in this study were relatively reliable.

### Functional Enrichment Analysis of Specific Organs

**FIGURE 5:**
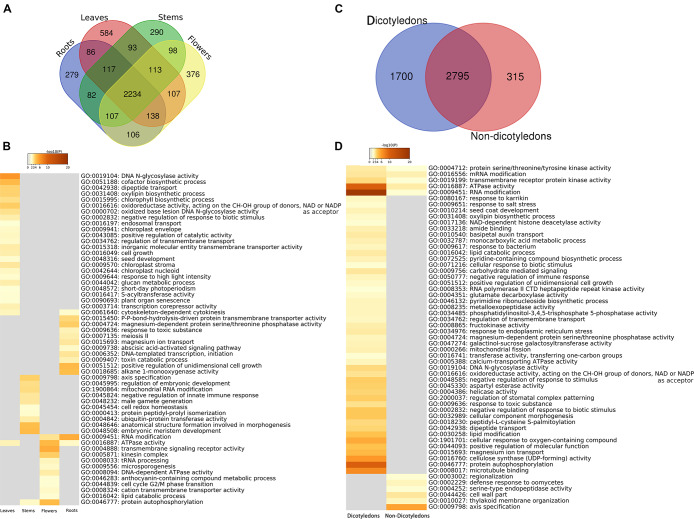
Organ-specific and dicotyledon-/non-dicotyledon-specific genes and functional enrichment analyses results. **(A)** Venn diagram of the annotated gene numbers in the leaves, roots, stems and flowers. **(B)** Heat map of *P*-values of significant enriched functional categories on each organ. The color key from gray to brown indicates large to small *P*-values. **(C)** Venn diagram of the annotated gene numbers in dicotyledons and non-dicotyledons. **(D)** Heat maps of *P*-values of significant enriched functional categories on dicotyledons or non-dicotyledons. The color key from gray to brown indicates large to small *P*-values.

There were 4,810 kinds of genes expressed in one or more organs, and 2,234 (44.46%) were shared by all four organs. The number of kinds of genes specific to organs is 279 for roots, 584 for leaves, 290 for stems, and 376 for flowers (Figure 5A and Supplementary Data S2/specific genes). Enrichment analysis was performed on genes exclusively expressed in only one organ by using Metascape. The results showed the organ-specific functional patterns on these four groups (Figure 5B and Supplementary Data S2/enriched GO terms), and some enriched items might be explained by knowledge of the function of the specific organ in the organisms. For example, in leaves, chloroplast envelope (GO:0009941), chloroplast stroma (GO:0009570), chloroplast nucleoid (GO:0042644), chlorophyll biosynthetic process (GO:0015995), response to high light intensity (GO:0009644), and short-day photoperiodism (GO:0048572) were detected. Chloroplast envelope, chloroplast stroma, chloroplast nucleoid, and chlorophyll biosynthetic process are related to chloroplast. The variation of light intensity has obvious effects on leaf external morphology, internal anatomy, and physiological characteristics (Feng et al., 2018). Photoperiod has effects on photosynthesis (Mousseau, 1984). In roots, a response to toxic substance (GO:0009636), abscisic acid (ABA)-activated signaling pathway (GO:0009738), and toxin catabolic process (GO:0009407) were detected. Plants are able to release chemical compounds from their roots into the soil. Some of these products are toxic when the roots of neighboring plants take them up (Bonner, 1950). As a result, the response to toxic substance and toxin catabolic process might be a “protective behavior” of roots. ABA, a major abiotic stress-responsive hormone, plays an important role in root hair elongation (Richardson et al., 2009; Wang T. et al., 2017). ABA enhances both auxin transport and auxin biosynthesis in root tips, and ABA and auxin co-regulate a set of genes to promote root hair length (Wang T. et al., 2017). In flowers, enriched function included microsporogenesis (GO:0009556) and an anthocyanin-containing compound metabolic process (GO:0046283). The relationship between microsporogenesis and flower development has been examined in some research. Generally, microsporogenesis and pollen formation are precisely timed and choreographed, and meiosis occurs in a precise chronological order that correlates with the flower bud size (Yang and Kang, 2015). Anthocyanins are commonly found in flowers and the fruits of many plants. Most of the red-, purple-, and blue-colored flowers contained anthocyanins (Khoo et al., 2017). Additionally, significant variation in one metabolite that belongs to “Vitamin” class was putatively identified as [5-Hydroxy-4-(hydroxymethyl)-6-methyl-3-pyridinyl]methyl dihydrogen phosphate in leaves rather than flowers (Sotelo-Silveira et al., 2015). Our results showed that genes mapped to the reference canonical pathways in KEGG with vitamin B6 metabolic function were only found in leaf-specific genes (AT5G53580). Very interestingly, seed development (GO:0048316) in leaves and male gamete generation (GO:0048232) in stems were also found in the results.

### Functional Enrichment Analysis of Dicotyledons and Non-dicotyledons

In this study, 5 of the samples were dicotyledons (hot1, hot3, hot6, hot8, and hot10); the other 15 samples (hot2, hot4, hot5, hot7, and hot9 and 10 cold-propertied samples) were non-dicotyledons. The specific genes of dicotyledons or non-dicotyledons were found. The number of specific genes in dicotyledons and non-dicotyledons were 1,700 and 315, respectively (Figure 5C and Supplementary Data S3/specific genes). Several GO functional categories were identified by enrichment analysis (Figure 5D and Supplementary Data S3/enriched GO terms). Among them, regulation of stomatal complex patterning (GO:2000037) was only enriched in dicotyledons. As eudicots were also known as the “three-pore pollen group,” because their pollen had three or more pores, which distinguished them from other angiosperms (Dunn and Campbell, 1965), this identified category might be related to the phenomenon that dicotyledons have more pores. The gene function enrichment analysis results in plant organs, dicotyledons, and non-dicotyledons suggests that our analysis strategy has a certain degree of credibility.

### Functional Enrichment Analysis and Comparison Between HMs With Cold and Hot Properties

**FIGURE 6:**
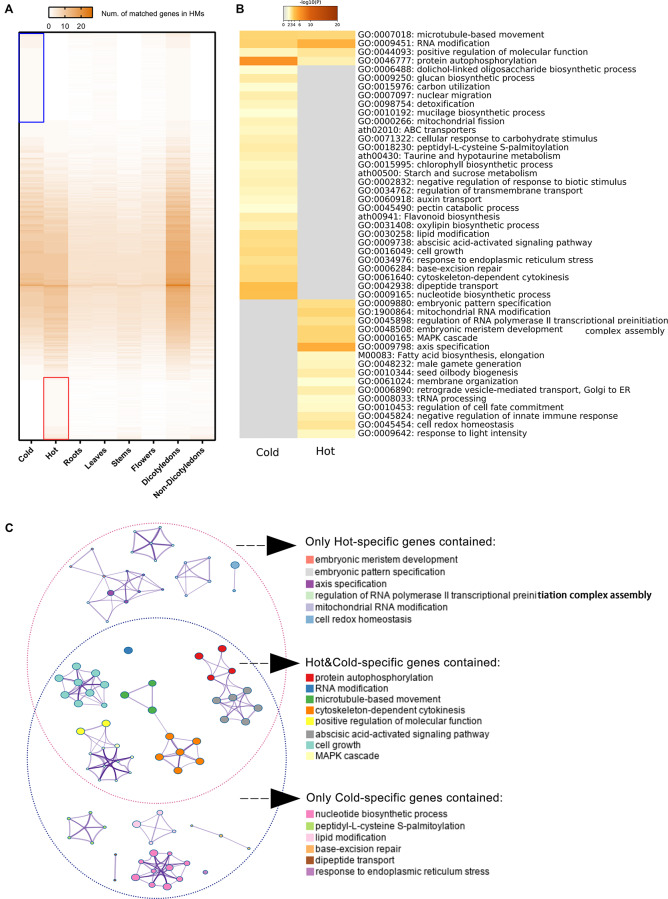
Specific genes of cold/hot HMs and functional enrichment analyses results. **(A)** Heat map of numbers matched genes in different sample groups. The color key from gray to brown indicates small to large number of matches. Blue/red box indicates the reference genes matched only genes in the cold/hot group when genes divided into two groups according to their cold or hot HMs. **(B)** Heat maps of *P*-values of significant enriched functional categories on cold and/or hot group. The color key from gray to brown indicates large to small *P*-values. **(C)** Network of enriched categories basing on hot/cold-specific genes. Each node represents an enriched category. Its size indicated the number of genes, for which the category contained a range of 3–63. The thickness of the edge indicated the degree of membership similarity between two nodes. The representative terms of the clusters are shown on the legend.

709 hot-specific and 1,039 cold-specific genes were identified, and the numbers of genes in different groups of HMs matched to reference genes were illustrated by heat map; the red and blue boxes indicate these hot-/cold-specific genes (Figure 6A). The complete list of cold or hot property-specific genes and the enriched functions was shown in Supplementary Data S4. The gene enrichment analysis results showed that five functional categories were shared among the cold- and hot-propertied HMs, such as protein autophosphorylation (GO:0046777), microtubule-based movement (GO:0007018), etc., meaning that different group-specific genes of these two groups belonged to the same categories that obtained the significant values through the analysis (Figure 6B). In the results, there were also several GO Biological processes and KEGG pathways identified as enriched categories only in the cold HMs or hot MHs. Among the enriched KEGG pathways, flavonoid biosynthesis (ath00941), ABC transporters (ath02010), taurine and hypotaurine metabolism (ath00430), and starch and sucrose metabolism (ath00500) were detected only in the cold HMs, and fatty acid biosynthesis and elongation (M00083) was only in the hot HMs. The searching results of chemical components of HMs and their metabolic pathways (Supplementary Data S5) indicated some links between group-specific chemical ingredients and group-specific enriched KEGG pathways. For example, among cold-specific chemical ingredients, chrysoeriol was shared in cold3 (*Radix Scutellariae*) and cold9 (*Flos Lonicerae*); swertiajaponin was shared in cold4 (*Herba Catharanthi Rosei*) and cold7 (*Radix Gentianae*); luteolin was shared in cold2 (*Herba Dracocephali Tangutici*), cold7 (*Radix Gentianae*), and cold9 (*Flos Lonicerae*); and paniculatin and wogonin were shared in cold3 (*Radix Scutellariae*) and cold5 (*Herba Andrographis*). These five components belonged to flavor biosynthesis (ath900941). In addition, inositol was shared in cold4 (*Herba Catharanthi Rosei*) and cold9 (*Flos Lonicerae*), which belong to ABC transporters (ath02010). Similarly, in hot-specific chemical ingredients, lauric acid and octanoic acid were shared in hot3 (*Rhizoma Curcumae Longae*) and hot6 (*Herba Ephedrae*); hexadecanoic acid and heptadecane were shared in hot6 (*Herba Ephedrae*) and hot9 (*Radix Notoginseng*); and pentadecanoic acid was shared in hot3 (*Rhizoma Curcumae Longae*), hot6 (*Herba Ephedrae*) and hot9 (*Radix Notoginseng*). These five components belonged to fatty acid biosynthesis and elongation (M00083).

The enriched candidate categories were clustered and further formed the networks of functional blocks (Figure 6C). The clusters with representative terms and the similarity links between the nodes provide a more comprehensive and higher-level visualization for the difference in gene function modules between cold and hot HMs. The terms of clusters belong to independent network blocks that only contained cold-specific genes were embryonic meristem development, embryonic pattern specification, axis specification, regulation of RNA polymerase II transcriptional preinitiation complex assembly, mitochondrial RNA modification, and cell redox homeostasis, while the terms deduced only by hot-specific genes were nucleotide biosynthetic process, peptidy-L-cysteine *S*-palmitoylation, lipid modification, base-excision repair, dipeptide transport, and response to endoplasmic reticulum stress.

## Discussion

Nowadays, theories in cold and hot properties of HMs have become a topic of interest and been researched from different aspects during these years. The relevant research can be divided into two categories based on their research objects. One is to study the physical or clinical responses (thermal behavior, anti-oxidative activity, etc.) of test subjects (Fu et al., 2015), and the other is to study the chemical materials of herbs themselves (Long et al., 2011). With the rapid development of the next-generation sequencing (NGS), RNA sequencing (RNA-Seq) is widely used in the analysis of transcriptomes of various organisms. For RNA-Seq, improved sequencing throughput and accuracy, shortened sequencing time, and significantly reduced price have opened the door to a better understanding of the sophisticated mechanisms of more species of plants. To date, our research makes the first attempt to study the differences between cold and hot HMs based on their gene expression profiles by taking original material of HMs as research object.

To study the gene function differences between different properties of HMs, cross-species comparison of plants was necessary and informative. Although there has been existing research on comparing gene expression among different species (Kristiansson et al., 2013; Shaar-Moshe et al., 2015), these methods did not seem to be suitable in this study for the less common (non-model species) and distantly related species of HMs. In this study, we established a method for the cross-species annotation of conserved genes by using transcriptome data of 20 HMs with cold and hot properties. As cross-species gene expression analyses were often hampered by the lack of publicly available genomes and gene orthology information, especially for species that were not common, using the operation procedures of BUSCO, we developed a pipeline for generating species-specific ortholog sets, profiled gene expression by RNA-seq, and identified the specific genes grouped by different features (organs, hot/cold properties, and dicotyledons/non-dicotyledons).

In order to ensure the reliability of the data sources, we applied strict selection procedures for choosing RNA-seq data included in current study. As HMs are specific parts of plants, which grow in specified areas with specific environment (Huang et al., 2011), all of the datasets were obtained from the exact samples for medicinal purpose. In terms of phylogenetic relationships, the 20 species of samples recruited were scattered within the lineage of plants from BUSCO set (Figure 4A). As for sequencing platform, all of 20 datasets were generated by Illumina paired-end sequencing with the similar read length. Literature searching and data screening results showed that the research of cold HMs is more popular (Figure 2C), which may be related to its high nephrotoxicity (Liang et al., 2013). Besides, the Illumina sequencing and transcriptome assembly results showed that there was no statistical difference in sequencing throughput, quality (Figures 3C,D), and assembly variables between cold and hot HMs, except for N50 (Figure 3E). These results suggest that the sequencing and assembly processes were unlikely to cause systemic bias in the follow-up analysis.

As for cross-species gene annotation, the complexity of genes on plant genomes and the incompleteness of gene annotation information makes it almost impossible to compare the expression of all genes across species, as plant genome sizes vary dramatically, ranging from 0.063 to 148.8 Gbp (Greilhuber et al., 2006; Hidalgo et al., 2017); meanwhile, the length of single-copy regions varies widely among plant species (Claros et al., 2012), and the protein-coding gene count is not significantly correlated to genome size (Michael, 2014). As a result, only the conserved genes of the 20 HMs inferred by *A. thaliana* were annotated and their information was deployed for enrichment analysis in the present study. Besides, relative to sensitivity, accuracy of the annotation was more concerned in this study, which was reflected in the followings. First, only the assembled transcripts with TPM greater than 1 were remained for annotation. Second, the cutoff for BLATSP was strict: 1e-114 for the closest species (cold10) and 1e-16 for the furthest species (hot6). Third, only a small percentage (1.73–5.30%) of unigenes of each HMs and a small percentage (17.54%) of genes on the reference genome matched each other established the links for gene annotation. It should be noted that the numbers of genes for calculation of enrichment parameters were the numbers of genes on reference genome. Thus, the different functional gene modules between groups were the results obtained through the gene function of specific parts of genomic mapped by conserved genes of HMs.

There were three and four types of organs from which cold and hot HMs were sampled, respectively (Figure 3C). This design was to prevent the difference between cold and hot HMs in the results due to organ specificity. The main purpose of performing enrichment analysis on each type of organs or dicotyledons/non-dicotyledons was to assess the effectiveness of the whole computational pipeline. Among the results, expressed genes in leaves were enriched in chloroplast- and chlorophyll-associated functional categories, expressed genes in roots were enriched in toxic and ABA-associated categories, and expressed genes in flower were enriched in microsporogenesis and anthocyanin-associated categories. Meanwhile, regulation of stomatal complex patterning was an enriched category found in dicotyledons. These findings match what we already know about the specific gene functions of plant organs or dicotyledons (see Results sections “Functional Enrichment Analysis of Specific Organs” and “Functional Enrichment Analysis of Dicotyledons and Non-dicotyledons”). Although seed development (GO:0048316) was found in leaves as well as male gamete generation (GO:0048232) in stems, which might be due to the types of organs that are not included the seed and the complex multi-function of genes, the results were still somewhat reasonable under the analysis strategy.

Some of natural products are produced by specific metabolic pathways of plants. These natural products can be served as drugs to modulate molecular networks of humans, which is probably because their corresponding biosynthesis pathways are chemico-biologically associated with the human molecular networks (Zhang B. et al., 2016). In our results, expressed genes in cold HMs were enriched in associated metabolic pathways of flavonoid, taurine, hypotaurine, starch, and sucrose, as well as a pathway for ABC transporters and genes of hot HMs enriched in the pathway of fatty acid biosynthesis and elongation. These were consistent with the metabolic pathways of cold/hot-specific chemical ingredients in plants (see Results sections “Functional Enrichment Analysis and Comparison Between HMs With Cold and Hot Properties”). The results suggested that there might be different patterns of compound metabolism between cold and hot HMs, which might play different roles in affecting the metabolic network of drug users. The Enrichment Network provides a more concise result with regards to the enrichment of expressed gene function (Figure 6C). For hot HMs, biological processes associated with embryonic development, mitochondrial RNA modification, cell redox homeostasis, and so on were extracted as the hot-specific functional modules. For cold HMs, processes associated with nucleotide biosynthetic and repair, peptidy-L-cysteine *S*-palmitoylation, lipid modification, dipeptide transport, and response to endoplasmic reticulum stress, were extracted as the cold-specific functional modules. The functional modules and their relationship with the hot/cold property of HMs need to be further verified and interpreted.

Here we describe, to the best of our knowledge, a comprehensive method for the comparison of RNA-seq data among plant species. The novelty should be emphasized. Although previous research has made an effort toward interspecies comparisons, an approach for distant species comparison has not been developed to date. Furthermore, the comparison of RNA-seq data among different species in the present study means that comparisons among huge numbers of species are possible. However, there remains a defect to be discussed. By analyzing suitable transcriptomes of only 10 cold and 10 hot propertied HMs, although the results of specific conserved gene expressing patterns could be obtained, and some could be found reasonable, it was very possible that recruiting more HMs would have impact on the results. Thus, a larger sample size was needed to reach more reliable signals about the difference of functional gene modules between cold and hot HMs.

## Data Availability Statement

The datasets generated for this study can be found in the NCBI Sequence Read Archive (SRA) database: SRR832972, SRR2915458, SRR3367956, SRR1271859, SRR1519324, SRR3951703, SRR924095, SRR1045129, SRR3591711, SRR1565773, SRR1609976, SRR5367856, SRR3928562, SRR3104394, SRR1867750, SRR1188607, SRR3233423, SRR1186931, SRR1032053, and SRR1438496.

## Author Contributions

HW launched ideas and designed the experiments. ArL performed most of the analysis. ArL and HW wrote the manuscript. HW revised the manuscript. AqL visualized the pipeline of cross-species gene annotation and selection of specific expressed genes in Figure 1. JG provided advice and supervised the work. All authors reviewed and approved the final version of the manuscript.

## Conflict of Interest

The authors declare that the research was conducted in the absence of any commercial or financial relationships that could be construed as a potential conflict of interest.

## Footnotes

1 http://busco.ezlab.org

2 www.orthodb.org

3 https://www.ncbi.nlm.nih.gov/pubmed

4 http://www.embase.com

5 http://scholar.google.cn/

6 https://www.ncbi.nlm.nih.gov/taxonomy/

7 https://www.ncbi.nlm.nih.gov/sra/

8 http://www.bioinformatics.babraham.ac.uk/projects/fastqc/

9 http://metascape.org

10 https://www.genome.jp/kegg/kegg2.html

## Supplementary Material

FIGURE S1 FastQC results of each sample before and after quality control. **(A1,B1,C1,D1)** Results of raw data. **(A2,B2,C2,D2)** Results of clean data. **(A1,A2)** Average quality scores by nt locations of reads. **(B1,B2)** Number distributions of reads in average quality scores. **(C1,C2)** Percentages of base N content by nt locations of reads. **(D1,D2)** Percentage distributions of sequence GC content, green for “pass,” yellow for “warn,” red for “fail”.

FIGURE S2 The length distribution of unigenes.

FIGURE S3 Distribution of sample gene expression level. Gene expression levels were measure by log10(TPM). The different colors of the curves represented different species. **(A)** Density of RNA-seq gene expression level. **(B)** Cumulative frequency of RNA-seq gene expression level.

TABLE S1 Summary of Chinese herbal medicines.

TABLE S2 Classification and sampling sources of 20 plants and reference species.

TABLE S3 Sample sequencing data quality evaluation.

TABLE S4 Statistics for *de novo* transcriptome assembly of each sample.

DATA S1 Cross-species gene expression analysis results.

DATA S2 Organ-specific genes and enriched GO terms.

DATA S3 Dicotyledon-specific genes and non-dicotyledon-specific genes and enriched GO terms.

DATA S4 HMs property-specific genes and enriched categories.

DATA S5 HMs property-specific compounds and the metabolic pathways.

## References

[B1] Bauer-Mehren A. (2013). Integration of genomic information with biological networks using Cytoscape. *Methods Mol. Biol.* 1021 37–61. 10.1007/978-1-62703-450-0-3 23715979

[B2] Bolger A. M. Lohse M. Usadel B. (2014). Trimmomatic: a flexible trimmer for Illumina sequence data. *Bioinformatics* 30 2114–2120. 10.1093/bioinformatics/btu170 24695404 PMC4103590

[B3] Bonner J. (1950). The role of toxic substances in the interactions of higher plants. *Bot. Rev.* 16 51–65. 10.1007/bf02879785

[B4] Cahan P. Ahmad A. M. Burke H. Fu S. Lai Y. Florea L (2005). List of lists-annotated (LOLA): a database for annotation and comparison of published microarray gene lists. *Gene* 360 78–82. 10.1016/j.gene.2005.07.008 16140476

[B5] Camacho C. Coulouris G. Avagyan V. Ma N. Papadopoulos J. Bealer K. (2009). BLAST+: architecture and applications. *BMC Bioinformatics* 10:421. 10.1186/1471-2105-10-421 20003500 PMC2803857

[B6] Campain A. Yang Y. H. (2010). Comparison study of microarray meta-analysis methods. *BMC Bioinformatics* 11:408. 10.1186/1471-2105-11-408 20678237 PMC2922198

[B7] Capella-Gutierrez S. Silla-Martinez J. M. Gabaldon T. (2009). TrimAl: a tool for automated alignment trimming in large-scale phylogenetic analyses. *Bioinformatics* 25 1972–1973. 10.1093/bioinformatics/btp348 19505945 PMC2712344

[B8] Chan K. (1995). Progress in traditional Chinese medicine. *Trends Pharmacol. Sci.* 16 182–187. 7652926 10.1016/s0165-6147(00)89019-7

[B9] Chen L. Zeng P. Zeng J. Pan J. Chen Q. Chen Y (2016). Isolation and identification of chemical constituents of *Lindera glauca*. *J. Jiangsu University (Medicine Edition)* 26 422–428.

[B10] Cheng C. Krishnakumar V. Chan A. P. Thibaud-Nissen F. Schobel S. Town CD (2017). Araport11: a complete reannotation of the *Arabidopsis thaliana* reference genome. *Plant J.* 89 789–804. 10.1111/tpj.13415 27862469

[B11] Claros M. G. Bautista R. Guerrero-Fernandez D. Benzerki H. Seoane P. Fernandez-Pozo N (2012). Why assembling plant genome sequences is so challenging. *Biology (Basel)* 1 439–459. 10.3390/biology1020439 24832233 PMC4009782

[B12] Dunn D. B. Campbell G. K. S. A. (1965). Stomatal patterns of dicotyledons and monocotyledons. *Am. Midl. Nat.* 74 185–195.

[B13] Eddy S. R. (2011). Accelerated profile HMM searches. *PLoS Comput. Biol.* 7:e1002195. 10.1371/journal.pcbi.1002195 22039361 PMC3197634

[B14] Feng L. Raza M. A. Li Z. Chen Y. Khalid M. Du J (2018). The influence of light intensity and leaf movement on photosynthesis characteristics and carbon balance of soybean. *Front. Plant Sci.* 9 1952. 10.3389/fpls.2018.01952 30687355 PMC6338029

[B15] Fu J. Pang J. Zhao X. Han J. (2015). The quantitative ideas and methods in assessment of four properties of chinese medicinal herbs. *Cell Biochem. Biophys.* 71 1307–1312. 10.1007/s12013-014-0349-y 25395193

[B16] Fu X. Mervin L. H. Li X. Yu H. Li J. Mohamad Zobir SZ (2017). Toward understanding the cold, hot, and neutral nature of chinese medicines using in silico Mode-of-Action analysis. *J. Chem. Inf. Model* 57 468–483. 10.1021/acs.jcim.6b00725 28257573

[B17] Fu X. J. Liu H. B. Wang P. Guan H. S. (2009). A study on the antioxidant activity and tissues selective inhibition of lipid peroxidation by saponins from the roots of *Platycodon grandiflorum*. *Am. J. Chin. Med.* 37 967–975. 10.1142/S0192415X09007375 19885956

[B18] Gao W. Sun H. X. Xiao H. Cui G. Hillwig M. L. Jackson A (2014). Combining metabolomics and transcriptomics to characterize tanshinone biosynthesis in *Salvia miltiorrhiza*. *BMC Genomics* 15:73. 10.1186/1471-2164-15-73 24467826 PMC3913955

[B19] Garg A. Agrawal L. Misra R. C. Sharma S. Ghosh S. (2015). Andrographis paniculata transcriptome provides molecular insights into tissue-specific accumulation of medicinal diterpenes. *BMC Genomics* 16:659. 10.1186/s12864-015-1864-y 26328761 PMC4557604

[B20] Grabherr M. G. Haas B. J. Yassour M. Levin J. Z. Thompson D. A. Amit I. (2011). Full-length transcriptome assembly from RNA-Seq data without a reference genome. *Nat. Biotechnol* 29 644–652. 10.1038/nbt.1883 21572440 PMC3571712

[B21] Greilhuber J. Borsch T. Muller K. Worberg A. Porembski S. Barthlott W (2006). Smallest angiosperm genomes found in lentibulariaceae, with chromosomes of bacterial size. *Plant Biol. (Stuttg)* 8 770–777. 10.1055/s-2006-924101 17203433

[B22] Groves R. A. Hagel J. M. Zhang Y. Kilpatrick K. Levy A. Marsolais F (2015). Transcriptome Profiling of Khat (Catha edulis) and *Ephedra sinica* reveals gene candidates potentially involved in Amphetamine-Type Alkaloid Biosynthesis. *PLoS One* 10:e119701. 10.1371/journal.pone.0119701 25806807 PMC4373857

[B23] Gu S. Pei J. (2017). Innovating chinese herbal medicine: from traditional health practice to scientific drug discovery. *Front. Pharmacol.* 8:381. 10.3389/fphar.2017.00381 28670279 PMC5472722

[B24] He L. Xu X. Li Y. Li C. Zhu Y. Yan H (2013). Transcriptome analysis of buds and leaves using 454 pyrosequencing to discover genes associated with the biosynthesis of active ingredients in *Lonicera japonica* Thunb. *PLoS One* 8:e62922. 10.1371/journal.pone.0062922 23638167 PMC3636143

[B25] Hidalgo O. Pellicer J. Christenhusz M. Schneider H. Leitch A. R. Leitch IJ (2017). Is there an upper limit to genome size? *Trends Plant Sci.* 22 567–573. 10.1016/j.tplants.2017.04.005 28506667

[B26] Huang L. Guo L. Ma C. Gao W. Yuan Q. (2011). Top-geoherbs of traditional Chinese medicine: common traits, quality characteristics and formation. *Front. Med.* 5 185–194. 10.1007/s11684-011-0141-y 21695624

[B27] Huang Q. Huang X. Deng J. Liu H. Liu Y. Yu K. (2016). Differential gene expression between leaf and rhizome in atractylodes lancea: a comparative transcriptome analysis. *Front. Plant Sci.* 7:348. 10.3389/fpls.2016.00348 27066021 PMC4811964

[B28] Huang Y. Yao P. Leung K. W. Wang H. Kong X. P. Wang L (2018). The Yin-Yang property of chinese medicinal herbs relates to chemical composition but not Anti-Oxidative activity: an illustration using Spleen-Meridian herbs. *Front. Pharmacol.* 9:1304. 10.3389/fphar.2018.01304 30498446 PMC6249273

[B29] Jia L. Zhao Y. Xing X. Wang J. Zou W. Li R. (2010). [Investigation of differences between cold and hot nature of Mahuang decoction and Maxing Shigan decoction based on cold/hot plate differentiating assay]. *Zhongguo Zhong Yao Za Zhi* 35 2741–2744. 21246831

[B30] Junier T. Zdobnov E. M. (2010). The Newick utilities: high-throughput phylogenetic tree processing in the UNIX shell. *Bioinformatics* 26 1669–1670. 10.1093/bioinformatics/btq243 20472542 PMC2887050

[B31] Katoh K. Standley D. M. (2013). MAFFT multiple sequence alignment software version 7: improvements in performance and usability. *Mol. Biol. Evol.* 30 772–780. 10.1093/molbev/mst010 23329690 PMC3603318

[B32] Khoo H. E. Azlan A. Tang S. T. Lim S. M. (2017). Anthocyanidins and anthocyanins: colored pigments as food, pharmaceutical ingredients, and the potential health benefits. *Food Nutr. Res.* 61:1361779. 10.1080/16546628.2017.1361779 28970777 PMC5613902

[B33] Ko K. M. Leon T. Y. Mak D. H. Chiu P. Y. Du Y Poon M. K. T. (2006). A characteristic pharmacological action of ‘Yang-invigorating’ Chinese tonifying herbs: enhancement of myocardial ATP-generation capacity. *Phytomedicine* 13 636–642. 10.1016/j.phymed.2006.02.007 16647252

[B34] Ko K. M. Mak D. H. Chiu P. Y. Poon M. K. (2004). Pharmacological basis of ‘Yang-invigoration’ in Chinese medicine. *Trends Pharmacol. Sci.* 25 3–6. 10.1016/j.tips.2003.11.002 14723971

[B35] Ko K. M. Leung H. Y. (2007). Enhancement of ATP generation capacity, antioxidant activity and immunomodulatory activities by Chinese Yang and Yin tonifying herbs. *Chin. Med.* 2:3. 10.1186/1749-8546-2-3 17386115 PMC1847515

[B36] Kristiansson E. Osterlund T. Gunnarsson L. Arne G. Larsson D. G. Nerman O. (2013). A novel method for cross-species gene expression analysis. *BMC Bioinformatics* 14:70. 10.1186/1471-2105-14-70 23444967 PMC3679856

[B37] Langmead B. Salzberg S. L. (2012). Fast gapped-read alignment with Bowtie 2. *Nat. Methods* 9 357–359. 10.1038/nmeth.1923 22388286 PMC3322381

[B38] Le HS Oltvai Z. N. Bar-Joseph Z. (2010). Cross-species queries of large gene expression databases. *Bioinformatics* 26 2416–2423. 10.1093/bioinformatics/btq451 20702396 PMC2944203

[B39] Li B. Dewey C. N. (2011). RSEM: accurate transcript quantification from RNA-Seq data with or without a reference genome. *BMC Bioinformatics* 12:323. 10.1186/1471-2105-12-323 21816040 PMC3163565

[B40] Li H. Fu Y. Sun H. Zhang Y. Lan X. (2017). Transcriptomic analyses reveal biosynthetic genes related to rosmarinic acid in *Dracocephalum tanguticum*. *Sci. Rep.* 7:74. 10.1038/s41598-017-00078-y 28250432 PMC5428373

[B41] Li M. J. Yang Y. H. Chen X. J. Wang F. Q. Lin W. X. Yi Y. J (2013). Transcriptome/degradome-wide identification of R. *Glutinosa* miRNAs and their targets: the role of miRNA activity in the replanting disease. *PLoS One* 8:e68531. 10.1371/journal.pone.0068531 23861915 PMC3702588

[B42] Li S. Zhang Z. Q. Wu L. J. Zhang X. G. Li Y. D. Wang Y. Y (2007). Understanding ZHENG in traditional Chinese medicine in the context of neuro-endocrine-immune network. *IET Syst. Biol.* 1 51–60. 10.1049/iet-syb:20060032 17370429

[B43] Li W. F. Jiang J. G. Chen J. (2008). Chinese medicine and its modernization demands. *Arch. Med. Res.* 39 246–251. 10.1016/j.arcmed.2007.09.011 18164973

[B44] Liang F. Li L. Wang M. Niu X. Zhan J. He X. (2013). Molecular network and chemical fragment-based characteristics of medicinal herbs with cold and hot properties from Chinese medicine. *J. Ethnopharmacol.* 148 770–779. 10.1016/j.jep.2013.04.055 23702041

[B45] Liu J. Hou J. Jiang C. Li G. Lu H. Meng F. (2015). Deep sequencing of the *scutellaria* baicalensis georgi transcriptome reveals flavonoid biosynthetic profiling and Organ-Specific gene expression. *Plos One* 10:e136397. 10.1371/journal.pone.0136397 26317778 PMC4552754

[B46] Liu M. Yang B. Cheung W. Yang K. Y. Zhou H. Sui-Lam Kwok J (2015). Transcriptome analysis of leaves, roots and flowers of Panax notoginseng identifies genes involved in ginsenoside and alkaloid biosynthesis. *BMC Genomics* 16:265. 10.1186/s12864-015-1477-5 25886736 PMC4399409

[B47] Liu Q. Wen C. Zhao H. Zhang L. Wang J. Wang Y. (2014). RNA-Seq reveals leaf cuticular Wax-Related genes in welsh onion. *PLoS one* 9:e113290. 10.1371/journal.pone.0113290 25415343 PMC4240658

[B48] Liu T. Li W. Y. Liu X. W. Qi C. M. Yuan Z. H. (2016). Chemical Constituents from the roots of *Lindera glauca* and their antitumor activity on four different Cancer cell lines. *Zhong Yao Cai* 39 1789–1792. 30204386

[B49] Liu Y. Wang Y. Guo F. Zhan L. Mohr T. Cheng P. (2017). Deep sequencing and transcriptome analyses to identify genes involved in secoiridoid biosynthesis in the Tibetan medicinal plant *Swertia mussotii*. *Sci. Rep.* 7:43108. 10.1038/srep43108 28225035 PMC5320516

[B50] Liu Z. Guo F. Wang Y. Li C. Zhang X. Li H. (2016). BATMAN-TCM: a bioinformatics analysis tool for molecular mechANism of traditional chinese medicine. *Sci. Rep.* 6. 21146 10.1038/srep21146 26879404 PMC4754750

[B51] Long W. Liu P. Xiang J. Pi X. Zhang J. Zou Z. (2011). A combination system for prediction of Chinese materia medica properties. *Comput. Methods Prog. Biomed.* 101 253–264. 10.1016/j.cmpb.2011.01.006 21315471

[B52] Matur F. Ulgen Y. (2019). 2D-ROC: a receiver operating surface and its illustrative application in clinical diagnostics. *Physiol. Meas.* 40:75004. 10.1088/1361-6579/ab2564 31141795

[B53] Michael T. P. (2014). Plant genome size variation: bloating and purging DNA. *Brief Funct. Genom.* 13 308–317. 10.1093/bfgp/elu005 24651721

[B54] Mousseau M. (1984). “Effects of photoperiod on photosynthesis,” in *Advances in Photosynthesis Research. Advances in Agricultural Biotechnology, vol 4.* ed Sybesma C. (Dordrecht: Springer)

[B55] Newman J. C. Weiner A. M. (2005). L2L: a simple tool for discovering the hidden significance in microarray expression data. *Genome Biol* 6:R81. 10.1186/gb-2005-6-9-r81 16168088 PMC1242216

[B56] Niu J. Chen Y. An J. Hou X. Cai J. Wang J (2015). Integrated transcriptome sequencing and dynamic analysis reveal carbon source partitioning between terpenoid and oil accumulation in developing *Lindera glauca* fruits. *Sci. Rep.* 5:15017. 10.1038/srep15017 26446413 PMC4597268

[B57] Provart N. J. Alonso J. Assmann S. M. Bergmann D. Brady S. M. Brkljacic J. (2016). 50 years of *Arabidopsis* research: highlights and future directions. *New Phytol.* 209 921–944. 10.1111/nph.13687 26465351

[B58] Rai A. Kamochi H. Suzuki H. Nakamura M. Takahashi H. Hatada T. (2017). De novo transcriptome assembly and characterization of nine tissues of *Lonicera japonica* to identify potential candidate genes involved in chlorogenic acid, luteolosides, and secoiridoid biosynthesis pathways. *J. Nat. Med.* 71 1–15. 10.1007/s11418-016-1041-x 27629269 PMC5214891

[B59] Ren Y. S. Wang J. B. Zhao Y. L. Zhang P. Zhao H. P. Zhang XR. (2009). [COLD and HOT nature of Coptis & Evodia and their prescriptions investigated with diet restriction/cold-water swimming mice models]. *Yao Xue Xue Bao* 44 1221–1227. 21355322

[B60] Richardson A. E. Barea J. McNeill A. M. Prigent-Combaret C. (2009). Acquisition of phosphorus and nitrogen in the rhizosphere and plant growth promotion by microorganisms. *Plant Soil* 321 305–339. 10.1007/s11104-009-9895-2

[B61] Sahoo A. Kar B. Sahoo S. Ray A. Nayak S. (2016). Transcriptome profiling of *Curcuma longa* L. Cv. Suvarna. *Genomics Data* 10 33–34. 10.1016/j.gdata.2016.09.001 27668184 PMC5024144

[B62] Shaar-Moshe L. Hubner S. Peleg Z. (2015). Identification of conserved drought-adaptive genes using a cross-species meta-analysis approach. *BMC Plant Biol.* 15:111. 10.1186/s12870-015-0493-6 25935420 PMC4417316

[B63] Slater G. S. Birney E. (2005). Automated generation of heuristics for biological sequence comparison. *BMC Bioinformatics* 6:31. 10.1186/1471-2105-6-31 15713233 PMC553969

[B64] Smith-Unna R. Boursnell C. Patro R. Hibberd J. M. Kelly S. (2016). TransRate: reference-free quality assessment of De novo transcriptome assemblies. *Genome Res.* 26 1134–1144. 10.1101/gr.196469.115 27252236 PMC4971766

[B65] Soetaert S. S. Van Neste C. M. Vandewoestyne M. L. Head S. R. Goossens A. Van Nieuwerburgh FC (2013). Differential transcriptome analysis of glandular and filamentous trichomes in *Artemisia annua*. *BMC Plant Biol.* 13:220. 10.1186/1471-2229-13-220 24359620 PMC3878173

[B66] Sotelo-Silveira M. Chauvin A. Marsch-Martínez N. Winkler R. de Folter S. (2015). Metabolic fingerprinting of *Arabidopsis thaliana* accessions. *Front. Plant Sci.* 6:365 10.3389/fpls.2015.00365 26074932 PMC4444734

[B67] Stamatakis A. (2014). RAxML version 8: a tool for phylogenetic analysis and post-analysis of large phylogenies. *Bioinformatics* 30 1312–1313. 10.1093/bioinformatics/btu033 24451623 PMC3998144

[B68] Stuart J. M. Segal E. Koller D. Kim S. K. (2003). A gene-coexpression network for global discovery of conserved genetic modules. *Science* 302 249–255. 10.1126/science.1087447 12934013

[B69] Su X. Li Q. Chen S. Dong C. Hu Y. Yin L. (2016). Analysis of the transcriptome of *Isodon rubescens* and key enzymes involved in terpenoid biosynthesis. *Biotechnol. Biotechnol. Equip.* 30 592–601. 10.1080/13102818.2016.1146086

[B70] Tsanakas G. F. Manioudaki M. E. Economou A. S. Kalaitzis P. (2014). De novo transcriptome analysis of petal senescence in *Gardenia jasminoides* Ellis. *BMC Genomics* 15:554. 10.1186/1471-2164-15-554 24993183 PMC4108791

[B71] Tseng G. C. Ghosh D. Feingold E. (2012). Comprehensive literature review and statistical considerations for microarray meta-analysis. *Nucleic Acids Res.* 40 3785–3799. 10.1093/nar/gkr1265 22262733 PMC3351145

[B72] Ung C. Y. Li H. Kong C. Y. Wang J. F. Chen Y. Z. (2007). Usefulness of traditionally defined herbal properties for distinguishing prescriptions of traditional Chinese medicine from non-prescription recipes. *J. Ethnopharmacol.* 109 21–28. 10.1016/j.jep.2006.06.007 16884871

[B73] Verma M. Ghangal R. Sharma R. Sinha A. K. Jain M. (2014). Transcriptome analysis of Catharanthus roseus for gene discovery and expression profiling. *PloS One* 9:e103583. 10.1371/journal.pone.0103583 25072156 PMC4114786

[B74] Wang H. Yuan X. Huang H. Zhang B. Cao C. Zhao HP (2017). Chemical constituents from *Swertia mussotii* Franch. *(Gentianaceae)*. *Nat. Prod. Res.* 31 1704–1708. 10.1080/14786419.2017.1286480 28278647

[B75] Wang T. Li C. Wu Z. Jia Y. Wang H. Sun S (2017). Abscisic acid regulates auxin homeostasis in rice root tips to promote root hair elongation. *Front. Plant Sci.* 8:1121. 10.3389/fpls.2017.01121 28702040 PMC5487450

[B76] Wang W. Wang Y. Zhang Q. Qi Y. Guo D. (2009). Global characterization of *Artemisia annua* glandular trichome transcriptome using 454 pyrosequencing. *BMC Genomics* 10:465. 10.1186/1471-2164-10-465 19818120 PMC2763888

[B77] Waterhouse R. M. Seppey M. Simão F. A. Manni M. Ioannidis P. Klioutchnikov G. (2018). BUSCO applications from quality assessments to gene prediction and phylogenomics. *Mol. Biol. Evol.* 35 543–548. 10.1093/molbev/msx319 29220515 PMC5850278

[B78] Wenping H. Yuan Z. Jie S. Lijun Z. Zhezhi W. (2011). De novo transcriptome sequencing in *Salvia miltiorrhiza* to identify genes involved in the biosynthesis of active ingredients. *Genomics* 98 272–279. 10.1016/j.ygeno.2011.03.012 21473906

[B79] Xiang Y. Haixia W. Zenggen L. Yanduo T. (2019). Anti-inflammatory activity of compounds isolated from Swertia mussotii. *Nat. Prod. Res.* 33 598–601. 10.1080/14786419.2017.1399385 29117731

[B80] Yang J. Kang X. (2015). Microsporogenesis and flower development in *Eucalyptus urophylla* x E. tereticornis. *Breed Sci.* 65 138–144. 10.1270/jsbbs.65.138 26069443 PMC4430506

[B81] Yang W. Fan C. Pei H. Ye W. Wang Y. (2016). HPLC-UV specific chromatogram for rhizomes of *Anemone flaccida*. *J. of Jinan University (Natural Science & Medicine Edition)* 37 277–284.

[B82] Yixi Q. Zhou J. Rezeng C. Limao C. (2020). Simultaneous determination of five flavonoids in *Dracocephalum tanguticum* with MSPE-HPLC. *Chin. J. Anal. Lab.* 39 379–384 10.13595/j.cnki.issn1000-0720.2019.070403

[B83] Yuan Y. Song L. Li M. Liu G. Chu Y. Ma L. (2012). Genetic variation and metabolic pathway intricacy govern the active compound content and quality of the Chinese medicinal plant *Lonicera japonica* thunb. *BMC Genomics* 13:195. 10.1186/1471-2164-13-195 22607188 PMC3443457

[B84] Zdobnov E. M. Tegenfeldt F. Kuznetsov D. Waterhouse R. M. Simão F. A. Ioannidis P (2017). OrthoDB v9.1: cataloging evolutionary and functional annotations for animal, fungal, plant, archaeal, bacterial and viral orthologs. *Nucleic Acids Res.* 45 D744–D749. 10.1093/nar/gkw1119 27899580 PMC5210582

[B85] Zeng S. Xiao G. Guo J. Fei Z. Xu Y. Roe BA. (2010). Development of a EST dataset and characterization of EST-SSRs in a traditional Chinese medicinal plant, *Epimedium sagittatum* (Sieb. Et Zucc.) Maxim. *BMC Genomics* 11:94. 10.1186/1471-2164-11-94 20141623 PMC2829513

[B86] Zhan C. Li X. Zhao Z. Yang T. Wang X. Luo B. (2016). Comprehensive analysis of the triterpenoid saponins biosynthetic pathway in anemone flaccida by transcriptome and proteome profiling. *Front. Plant Sci.* 7:1094. 10.3389/fpls.2016.01094 27504115 PMC4958654

[B87] Zhang B. Fu Y. Huang C. Zheng C. Wu Z. Zhang W. (2016). New strategy for drug discovery by large-scale association analysis of molecular networks of different species. *Sci. Rep.* 6:21872. 10.1038/srep21872 26912056 PMC4766474

[B88] Zhang G. Jiang N. Song W. Ma C. Yang S. Chen J. W (2016). De novo sequencing and transcriptome analysis of pinellia ternata identify the candidate genes involved in the biosynthesis of benzoic acid and ephedrine. *Front. Plant Sci.* 7:1209. 10.3389/fpls.2016.01209 27579029 PMC4986801

[B89] Zhang L. T. Takaishi Y. Zhang Y. W. Duan H. Q. (2008). Studies on chemical constituents from rhizome of *Anemone flaccida*. *Zhongguo Zhong Yao Za Zhi* 33 1696–1699. 18841769

[B90] Zhang W. Wei X. Meng H. Ma C. Jiang N. Zhang G.-H, et al. (2015). Transcriptomic comparison of the self-pollinated and cross-pollinated flowers of Erigeron breviscapus to analyze candidate self-incompatibility-associated genes. *BMC Plant Biol.* 15:248. 10.1186/s12870-015-0627-x 26463824 PMC4604739

[B91] Zhang X. Allan A. Li C. Wang Y. Yao Q. (2015). De novo assembly and characterization of the transcriptome of the chinese medicinal herb, gentiana rigescens. *Inter. J. Mol. Sci.* 16 11550–11573. 10.3390/ijms160511550 26006235 PMC4463717

[B92] Zhao X. L. Han J. X. (2013). The connotation of the quantum traditional Chinese medicine and the exploration of its experimental technology system for diagnosis. *Drug Discov. Ther.* 7 225–232. 10.5582/ddt.2013.v7.6.225 24423653

[B93] Zhao Y. L. Wang J. B. Xiao X. H. Zhao H. P. Zhou C. P. Zhang X. (2011). Study on the cold and hot properties of medicinal herbs by thermotropism in mice behavior. *J. Ethnopharmacol.* 133 980–985. 10.1016/j.jep.2010.09.014 20883763

[B94] Zhou Y. Kang L. Liao S. Pan Q. Ge X. Li Z. (2015). Transcriptomic analysis reveals differential gene expressions for cell growth and functional secondary metabolites in induced autotetraploid of chinese woad (Isatis indigotica fort.). *PLoS One* 10:e116392. 10.1371/journal.pone.0116392 25739089 PMC4349453

[B95] Zhou Y. Zhou B. Pache L. Chang M. Khodabakhshi A. H. Tanaseichuk O. (2019). Metascape provides a biologist-oriented resource for the analysis of systems-level datasets. *Nat. Commun.* 10:1523 10.1038/s41467-019-09234-6 30944313 PMC6447622

[B96] Zuo M. Yang C. Tian Q. Luo Y. Yang C. Liu J. H (2015). Chemical constituents of Dracoce-phalumtanguticum Maxim of genus *Dracocephalum*. *J. Yunnan Minzu University (Natural Sciences Edition)* 24 101–103.

