# Supplementary material for: Cross-Species Annotation of Expressed Genes and Detection of Different Functional Gene Modules Between 10 Cold- and 10 Hot-Propertied Chinese Herbal Medicines
Source: Front Genet. 2020 Jun 18;11:532. doi: 10.3389/fgene.2020.00532 (PMC7314971; doi:10.3389/fgene.2020.00532)
Supplement: FIGURE S1 — FastQC results of each sample before and after quality control. (A1,B1,C1,D1) Results of raw data. (A2,B2,C2,D2) Results of clean data. (A1,A2) Average quality scores by nt locations of reads. (B1,B2) Number distributions of reads in average quality scores. (C1,C2) Percentages of base N content by nt locations of reads. (D1,D2) Percentage distributions of sequence GC content, green for “pass,” yellow for “warn,” red for “fail”. [file Table_6.DOCX]

**Table S1 Summary of Chinese herbal medicines**

| **Family** | **Species** | **Organs** | **HM Chinese name** | **HM Latin name** | **Property** |
| --- | --- | --- | --- | --- | --- |
| Liliaceae | *Allium fistulosum* L. | Bulbs | Cong Bai^1^ | *Bulbus Allii Fistulosi* | warm |
|  |  | Inflorescences | Cong Hua^2^ | *Flos Allii Fistulosi* | warm |
|  |  | Roots | Cong Xu^1^ | *Radix Allii Fistulosi* | neutral |
|  |  | Leaves | Cong Ye^1, 3^ | *Folium Allii Fistulosi* | warm |
|  | *Allium sativum* L. | Bulbs | Da Suan^4^ | *Bulbus Allii Sativi* | warm |
|  | *Tulipa edulis* (Miq.) Baker | Stolons | Guang Ci Gu^5^ | *Bulbus Tulipae Edulis* | cold |
|  | *Polygonatum sibiricum* Red. | Rhizomes | Huang Jing^6^ | *Rhizoma Polygonati* | neutral |
| Lamiaceae | *Salvia miltiorrhiza* Bunge | Roots, Stems | Dan Shen^7-12^ | *Radix Salviae Miltiorrhizae* | cold |
|  | *Rabdosia rubescens* (Hemsl.) Hara | Leaves | Dong Ling Cao^13, 14^ | *Herba Rabdosiae Rubescentis* | warm |
|  | *Scutellaria baicalensis* Georgi | Roots | Huang Qin^15^ | *Radix Scutellariae* | cold |
|  | *Dracocephalum tanguticum* Maxim. | Leaves, Roots, Stems | Tang Gu Te Qing Lan^16^ | *Herba Dracocephali Tangutici* | cold |
| Euphorbiaceae | *Euphorbia fischeriana* Steud. | Roots | Bai Lang Du^17^ | *Radix Euphorbiae Fischerianae* | cold |
| Aquifoliaceae | *Ilex asprella* | Roots | Gang Mei Gen^18^ | *Radix Ilicis Asprella* | cool |
| Leguminosae | *Cassia angustifolia* Vahl. | Leaves | Fan Xie Ye^19^ | *Folium Sennae* | cool |
|  | *Glycyrrhiza uralensis* Fisch. | Roots | Gan Cao^20, 21^ | *Radix Glycyrrhizae* | neutral |
|  | *Pueraria lobata* (Willd.) Ohwi | Roots | Ge Gen^22, 23^ | *Radix Puerariae* | cool |
|  |  | Stems | Ge Man^22^ | *Caulis Puerariae* | cold |
|  |  | Leaves | Ge Ye^22, 23^ | *Folium Puerariae* | cool |
|  | *Astragalus membranaceus* | Roots | Huang Qi^24-27^ | *Radix Astragali* | warm |
|  | *Vigna radiata* L. Wilczek. | Seeds | Lv Dou^28^ | *Semen Vigna Radiata* | cold |
|  |  | Leaves | Lv Dou Ye^29^ | *Folium Vigna Radiata* | cold |
|  | *Ammopipanthus mongolicus* (Maxim. ex Kom.) Cheng f. | Leaves | Sha Dong Qing^30^ | *Caulis et Folium Ammopipanthis* | warm |
| Eucommiaceae | *Eucommia ulmoides* Oliv. | Leaves | Du Zhong Ye^31^ | *Folium Eucommiae* | warm |
| Taxaceae | *Taxus cuspidate* Sieb.et Zucc | Needles | Zi Shan^32^ | *Ramulus Taxi Cuspidatae* | neutral |
| Elaeagnaceae | *Hippophae rhamnoides* L. | Seeds | Sha Ji^33^ | *Fructus Hippophae* | warm |
| Cucurbitaceae | *Gynostemma pentaphyllum* (Thunb.) Makino | Leaves, Roots, Stems | Jiao Gu Lan^34^ | *Herba Gynostemmatis Pentaphylli* | cool |
|  | *Siraitia grosvenorii* | Fruits | Luo Han Guo^35^ | *Fructus Momordicae* | cool |
|  | *Luffa cylindrica* (L.) Roem. | Fruits | Si Gua^36, 37^ | *Fructus Luffae* | cool |
| Apocynaceae | *Catharanthus roseus* (L.) G. Don | Leaves, Roots, Stem, Flowers | Chang Chun Hua^38-44^ | *Herba Catharanthi Rosei* | cold |
| Zingiberaceae | *Curcuma longa* L. | Rhizomes | Jiang Huang^45-47^ | *Rhizoma Curcumae Longae* | warm |
| Violaceae | *Viola tricolor L.* | Leaves | San Se Jin^48^ | *Herba Violae Tricoloris* | cold |
| Asteraceae | *Xanthium strumarium* L. | Leaves | Cang Er^49^ | *Herba Xanthii* | cold |
|  | *Atractylodes lancea* (Thunb.) DC. | Rhizomes | Cang Zhu^50, 51^ | *Rhizoma Atractylodis* | warm |
|  | *Pyrethrum cinerariifolium* Trev. | Leaves, Flowers | Chu Chong Ju^52^ | *Herba Et Flos Pyrethri Cinerariifolii* | cool |
|  | *Erigeron breviscapus* (Vant.) Hand. -Mazz. | Whole plantlets | Deng Zhan Xi Xin^53-55^ | *Herba Erigerontis* | warm |
|  | *Carthamus tinctorius* L. | Petals | Hong Hua^56, 57^ | *Flos Carthami* | warm |
|  | *Dendranthema nankingense* (Hand.-Mazz.) | Leaves, stems | Ju Hua Nao^58-60^ | *Caulis et Folium Dendranthemae* | cool |
|  | *Dendranthema morifolium* (Ramat.) Tzvel. | Leaves | Ju Hua Ye^61^ | *Folium Chrysanthemi* | neutral |
|  | *Artemisia annua* L. | Leaves, Flowers buds | Qing Hao^62-64^ | *Herba Artemisiae Annuae* | cold |
| Acanthaceae | *Andrographis paniculata* (Burm. f.) Nees | Leaves, Roots | Chuan Xin Lian^65, 66^ | *Herba Andrographis* | cold |
|  | *Baphicacanthus cusia* (Nees) Bremek. | Roots | Nan Ban Lan Gen^67^ | *Rhizoma Et Radix Baphicacanthis Cusiae* | cold |
| Orchidaceae | *Nervilia fordii* (Hance) Schltr. | Leaves, Corm | Qing Tian Kui^68^ | *Herba Nerviliae Plicatae* | cool |
|  | *Dendrobium nobile* Lindl. | Stems | Shi Hu^69-75^ | *Herba Dendrobii* | cold |
|  | *Gastrodia elata* Bl. | Vegetative propagation corms and juvenile tubers | Tian Ma^76, 77^ | *Rhizoma Gastrodiae* | neutral |
| Nyssaceae | *Camptotheca acuminata* Decne. | Leaves | Xi Shu Ye^78^ | *Folium Camptothecae Acuminatae* | cold |
| Polygonaceae | *Polygonum multiflorum* Thunb. | Roots | He Shou Wu^79, 80^ | *Radix Polygoni Multiflori* | warm |
|  | *Polygonum cuspidatum* Sieb. et Zucc. | Roots | Hu Zhang^81^ | *Rhizoma Polygoni Cuspidati* | cold |
|  | *Fagopyrum dibotrys* (D. Don) Hara | Rhizomes | Jin Qiao Mai^82^ | *Rhizoma Fagopyri Dibotryis* | cold |
| Gentianaceae | *Swertia mussotii* Franch. | Leaves, Roots, Stems, Flowers | Zang Yin Chen^83^ | *Herba Swertiae Mussotii* | cold |
|  | *Gentiana rigescens* Franch. ex Hemsl. | Roots | Long Dan^84^ | *Radix Gentianae* | cold |
| Asclepiadaceae | *Marsdenia tenacissima* (Roxb.) Wight et Arn. | Mixture of leaves and stems | Tong Guang San^85^ | *Caulis Marsdeniae Tenacissimae* | cold |
| Ephedraceae | *Ephedra sinica* Stapf | Stems | Ma Huang^86, 87^ | *Herba Ephedrae* | warm |
|  |  | Roots | Ma Huang Gen | *Radix Ephedrae* | neutral |
| Ranunculaceae | *Anemone flaccida* Fr. Shmidt | Stems | Di Wu^88^ | *Rhizoma Anemones Flaccidae* | warm |
| Rubiaceae | *Gardenia jasminoides* Ellis | Petals | Zhi Zi Hua^89^ | *Flos Gardeniae Jasminoidis* | cold |
|  |  | Leaves | Zhi Zi Ye^90^ | *Folium Gardeniae Jasminoidis* | cold |
| Rosaceae | *Crataegus pinnatifida* Bunge | Fruits | Shan Zha^91, 92^ | *Fructus Crataegi* | warm |
| Solanaceae | *Physalis peruviana* L. | Leaves, Pooled | Deng Long Cao^93^ | *Herba Physalis* | cool |
|  | *Lycium chinense* Mill. | Leaves | Gou Qi Ye^94-96^ | *Folium Et Ramulus Lycii* | cool |
|  | *Lycium barbarum* L. | Fruits | Gou Qi Zi^96, 97^ | *Fructus Lycii* | neutral |
| Caprifoliaceae | *Lonicera japonica* Thunb. | Flowers | Jin Yin Hua^98, 99^ | *Flos Lonicerae* | cold |
|  |  | Stems | Ren Dong Teng^99^ | *Caulis Lonicerae* | cold |
|  | *Lonicera macranthoides* Hand.-Mazz. | Flowers | Shan Yin Hua^100^ | *Flos Lonicerae* | cold |
| Thymelaeaceae | *Aquilaria sinensis* (Lour.) Gilg | Stems | Chen Xiang^101-103^ | *Lignum Aquilariae Resinatum* | warm |
| Saururaceae | *Houttuynia cordata* Thunb. | Mixture of rhizome, stem, leaves and flowers | Yu Xing Cao^104^ | *Herba Houttuyniae* | cold |
| Apiaceae | *Bupleurum chinense* (Thorowax) | Roots | Chai Hu^105^ | *Radix Bupleuri* | cold |
|  | *Ligusticum Chuanxiong* | Rhizome | Chuan Xiong^106^ | *Rhizoma Chuanxiong* | warm |
|  |  | Leaves | Mi Wu^106^ | *Caulis et Folium Chuanxiong* | warm |
|  | *Angelica Sinensis* diels | Heads and tails | Dang Gui^107^ | *Radix Angelicae Sinensis* | warm |
|  | *Centella asiatica* | Leaves | Ji Xue Cao^108, 109^ | *Herba Centellae* | cold |
|  | *Oenanthe javanica* (BI.) DC | Leaves | Shui Qin^110^ | *Herba Oenanthes Javanicae* | cool |
| Moraceae | *Cannabis sativa* | Roots | Ma Gen^111^ | *Radix Cannabis* | neutral |
| Ranunculaceae | *Paeonia suffruticosa* | Petals | Mu Dan Hua^112-114^ | *Flos Moutan* | neutral |
|  |  | Roots | Mu Dan Pi^115^ | *Cortex Moutan* | cold |
| Brassicaceae | *Isatis indigotica* Fort. | Hairy Roots | Ban Lan Gen^116^ | *Radix Indigotica* | cold |
|  |  | Leaves | Qing Dai^117^ | *Indigo Naturalis* | cold |
|  | *Raphanus sativus* | Roots | Lai Fu^118-120^ | *Radix Raphani* | cool |
|  |  | Leaves | Lai Fu Ye^120-123^ | *Folium Raphani Sativi* | neutral |
| Punicaceae | *Punicai granatum* L. | Flowers buds | Shi Liu Hua^124^ | *Flos Granati* | neutral |
|  |  | Fruit Peel | Shi Liu Pi^125^ | *Pericarpium Granati* | warm |
| Huperziaceae | *Phlegmariurus carinatus* | Mixture of roots, stems and leaves | Da Shen Jin Cao^126^ | *Herba Phlegmariuri* | warm |
|  | *Huperzia serrata* | Leaves, Roots, Stems, Sporangia | Qian Ceng Ta^127, 128^ | *Herba Huperziae* | neutral |
| Caryophyllaceae | *Pseudostellaria heterophylla* (Miq.) Pax | Roots | Tai Zi Shen^129, 130^ | *Radix Pseudostellariae* | cold |
| Guttiferae | *Hypericum perforatum* L. (St. John's wort) | Mixture of roots, stems, leaves and flowers | Guan Ye Lian Qiao^131^ | *Herba Hyperici Perforati* | neutral |
| Araceae | *Pinellia ternate* (Thunb.) Berit | Tubers | Ban Xia^132^ | *Rhizoma Pinelliae* | warm |
|  | *Amorphophallus konjac* | Corms | Mo Yu^133^ | *Rhizoma Amorphophalli Rivieri* | cold |
| Sapindaceae | *Litchi chinensis* Sonn | Fruits | Li Zhi^133^ | *Fructus Litchi* | warm |
| Araliaceae | *Panax ginseng* | Roots | Ren Shen^136, 137^ | *Radix Ginseng* | warm |
|  |  | Flowers | Ren Shen Hua^137^ | *Flos Ginseng* | cold |
|  |  | Stems | Ren Shen Lu^137^ | *Rhizome Ginseng* | warm |
|  |  | Leaves | Ren Shen Ye^137^ | *Folium Ginseng* | cold |
|  | *Panax notoginseng* (Burk.) F.H. Chen ex C.Chow | Roots | San Qi^138-140^ | *Radix Notoginseng* | warm |
|  |  | Flowers | San Qi Hua^139^ | *Flos Notoginseng* | cool |
|  |  | Leaves | San Qi Ye^139^ | *Folium Notoginseng* | warm |
|  | *Panax Quinquefolium* | Roots | Xi Yang Shen^141, 142^ | *Radix Panacis Quinquefolii* | cold |
|  | *Panax japonicus* | Rhizomes | Zhu Jie Shen^143, 144^ | *Rhizoma Panacis Japonici* | warm |
|  |  | Leaves | Zhu Jie Ren Shen Ye^144^ | *Folium Panacis Japonici* | cold |
| Schisandraceae | *Schisandra chinensis* Turcz. (Baill.) | Fruits | Bei Wu Wei Zi^145^ | *Fructus Schisandrae Chinensis* | warm |
| Amaranthaceae | *Achyranthes bidentata* Blume | Roots | Niu Xi^146^ | *Radix Achyranthis Bidentatae* | neutral |
|  |  | Leaves | Niu Xi Jing Ye^146^ | *Caulis Et Folium Achyranthis* | neutral |
| Berberidaceae | *Epimedium sagittatum* (Sieb. et Zucc.) Maxim. | Leaves | Yin Yang Huo^147^ | *Herba Epimedii* | warm |
| Scrophulariaceae | *Rehmannia glutinosa* (Gaertn.) Libosch. | Leaves | Di Huang Ye^148, 149^ | *Folium Rehmanniae* | cold |
|  |  | Roots | Xian Di Huang^148-151^ | *Radix Rehmanniae* | cold |
|  | *Digitalis purpurea* | Leaves | Yang Di Huang^152^ | *Folium Digitalis* | warm |
| Ginkgoaceae | *Ginkgo biloba* | Seeds | Bai Guo^153^ | *Semen Ginkgo* | neutral |
|  |  | Leaves | Bai Guo Ye^154^ | *Folium Ginkgo* | neutral |
| Papaveraceae | *Macleaya cordata* | Roots, Leaves | Bo Luo Hui^155, 156^ | *Herba Macleayae Cordatae* | cold |
| Iridaceae | *Crocus sativus* | Stigma | Fan Hong Hua^157^ | *Stigma Croci* | neutral |
| Polygalaceae | *Polygala tenuifolia* | Roots | Yuan Zhi^158^ | *Radix Polygalae* | warm |
| Lauraceae | *Lindera glauca* (Sieb. et Zucc.) Bl | Fruits | Shan Hu Jiao^159^ | *Fructus Linderae* | cold |
|  |  | Roots | Shan Hu Jiao Gen^159^ | *Radix Linderae* | warm |

**References**

1. Abdelrahman, M. et al. RNA-sequencing-based transcriptome and biochemical analyses of steroidal saponin pathway in a complete set of Allium fistulosum—A. cepa monosomic addition lines. *PLOS ONE* **12**, e0181784 (2017).

2. Liu, Q. et al. Transcriptome Sequencing Analyses between the Cytoplasmic Male Sterile Line and Its Maintainer Line in Welsh Onion (Allium fistulosum L.). *International Journal of Molecular Sciences* **17**, 1058 (2016).

3. Liu, Q. et al. RNA-Seq Reveals Leaf Cuticular Wax-Related Genes in Welsh Onion. *PLoS ONE* **9**, e113290 (2014).

4. Sun, X., Zhou, S., Meng, F. & Liu, S. De novo assembly and characterization of the garlic (Allium sativum) bud transcriptome by Illumina sequencing. *Plant Cell Reports* **31**, 1823-1828 (2012).

5. Miao, Y. et al. Transcriptome Analysis of Differentially Expressed Genes Provides Insight into Stolon Formation in Tulipa edulis. *Frontiers in Plant Science* **7** (2016).

6. Wang, S. et al. De Novo Assembly and Analysis of Polygonatum sibiricum Transcriptome and Identification of Genes Involved in Polysaccharide Biosynthesis. *International Journal of Molecular Sciences* **18**, 1950 (2017).

7. Gao, W. et al. Combining metabolomics and transcriptomics to characterize tanshinone biosynthesis in Salvia miltiorrhiza. *BMC Genomics* **15**, 73 (2014).

8. Xu, Z. et al. Full-length transcriptome sequences and splice variants obtained by a combination of sequencing platforms applied to different root tissues ofSalvia miltiorrhiza and tanshinone biosynthesis. *The Plant Journal* **82**, 951-961 (2015).

9. Yang, L. et al. Transcriptome analysis of medicinal plant Salvia miltiorrhiza and identification of genes related to tanshinone biosynthesis. *PLoS One* **8**, e80464 (2013).

10. Zhou, W. et al. Comprehensive transcriptome profiling of Salvia miltiorrhiza for discovery of genes associated with the biosynthesis of tanshinones and phenolic acids. *Scientific Reports* **7** (2017).

11. Zhang, X. et al. Genome-wide characterisation and analysis of bHLH transcription factors related to tanshinone biosynthesis in Salvia miltiorrhiza. *Scientific Reports* **5** (2015).

12. Li, Y et al. Transcriptome characterization for Salvia miltiorrhiza using 454 GS FLX. *Yao Xue Xue Bao*, 524-529 (2010).

13. Su, X. et al. Analysis of the transcriptome of Isodon rubescens and key enzymes involved in terpenoid biosynthesis. **30**, 592 - 601 (2016).

14. Jin, B. et al. Functional Diversification of Kaurene Synthase-Like Genes inIsodon rubescens. *Plant Physiology* **174**, 943-955 (2017).

15. Liu, J. et al. Deep Sequencing of the Scutellaria baicalensis Georgi Transcriptome Reveals Flavonoid Biosynthetic Profiling and Organ-Specific Gene Expression. *PLOS ONE* **10**, e0136397 (2015).

16. Li, H., Fu, Y., Sun, H., Zhang, Y. & Lan, X. Transcriptomic analyses reveal biosynthetic genes related to rosmarinic acid in Dracocephalum tanguticum. *Scientific Reports* **7** (2017).

17. Barrero, R.A. et al. De novo assembly of Euphorbia fischeriana root transcriptome identifies prostratin pathway related genes. *BMC Genomics* **12**, 600 (2011).

18. Zheng, X., Xu, H., Ma, X., Zhan, R. & Chen, W. Triterpenoid Saponin Biosynthetic Pathway Profiling and Candidate Gene Mining of the Ilex asprella Root Using RNA-Seq. *International Journal of Molecular Sciences* **15**, 5970-5987 (2014).

19. Rama Reddy, N.R. et al. Next Generation Sequencing and Transcriptome Analysis Predicts Biosynthetic Pathway of Sennosides from Senna (Cassia angustifolia Vahl.), a Non-Model Plant with Potent Laxative Properties. *PLOS ONE* **10**, e0129422 (2015).

20. Ramilowski, J.A. et al. Glycyrrhiza uralensis Transcriptome Landscape and Study of Phytochemicals. *Plant and Cell Physiology* **54**, 697-710 (2013).

21. Tamura, K. et al. CYP716A179 functions as a triterpene C-28 oxidase in tissue-cultured stolons of Glycyrrhiza uralensis. *Plant Cell Reports* **36**, 437-445 (2017).

22. Han, R. et al. Transcriptomic landscape of Pueraria lobata demonstrates potential for phytochemical study. *Frontiers in Plant Science* **6** (2015).

23. Wang, X., Li, S., Li, J., Li, C. & Zhang, Y. De novo transcriptome sequencing in Pueraria lobata to identify putative genes involved in isoflavones biosynthesis. *Plant Cell Reports* **34**, 733-743 (2015).

24. Li, J. et al. Long read reference genome-free reconstruction of a full-length transcriptome from Astragalus membranaceus reveals transcript variants involved in bioactive compound biosynthesis. *Cell Discovery* **3**, 17031 (2017).

25. Tuan, P.A. et al. Transcriptional Profiling and Molecular Characterization of Astragalosides, Calycosin, and Calycosin-7-O-β-d -glucoside Biosynthesis in the Hairy Roots ofAstragalus membranaceus in Response to Methyl Jasmonate. *Journal of Agricultural and Food Chemistry* **63**, 6231-6240 (2015).

26. Chen, J. et al. Global transcriptome analysis profiles metabolic pathways in traditional herb Astragalus membranaceus Bge. var. mongolicus (Bge.) Hsiao. *BMC Genomics* **16 Suppl 7**, S15 (2015).

27. Jia, X. et al. Integrating transcriptomics and metabolomics to characterise the response of Astragalus membranaceus Bge. var. mongolicus (Bge.) to progressive drought stress. *BMC Genomics* **17** (2016).

28. Tian, X., Li, S., Liu, Y. & Liu, X. Transcriptomic Profiling Reveals Metabolic and Regulatory Pathways in the Desiccation Tolerance of Mungbean (Vigna radiata [L.] R. Wilczek). *Frontiers in Plant Science* **7** (2016).

29. Moe, K.T. et al. Sequence information on simple sequence repeats and single nucleotide polymorphisms through transcriptome analysis of mungbean. *J Integr Plant Biol* **53**, 63-73 (2011).

30. Pang, T. et al. Characterization of the Transcriptome of the Xerophyte Ammopiptanthus mongolicus Leaves under Drought Stress by 454 Pyrosequencing. *PLOS ONE* **10**, e0136495 (2015).

31. Wang, W. & Zhang, X. Identification of the Sex-Biased Gene Expression and Putative Sex-Associated Genes in Eucommia ulmoides Oliver Using Comparative Transcriptome Analyses. *Molecules* **22**, 2255 (2017).

32. Wu, Q. et al. Transcriptome analysis of Taxus cuspidata needles based on 454 pyrosequencing. *Planta Med* **77**, 394-400 (2011).

33. Fatima, T. et al. Fatty acid composition of developing sea buckthorn (Hippophae rhamnoides L.) berry and the transcriptome of the mature seed. *PLoS One* **7**, e34099 (2012).

34. Chen, Q. et al. Transcriptome Sequencing ofGynostemma pentaphyllum to Identify Genes and Enzymes Involved in Triterpenoid Biosynthesis. *International Journal of Genomics* **2016**, 1-10 (2016).

35. Tang, Q. et al. An efficient approach to finding Siraitia grosvenorii triterpene biosynthetic genes by RNA-seq and digital gene expression analysis. *BMC Genomics* **12**, 343 (2011).

36. Chen, X. et al. Genome-wide transcriptome profiling reveals novel insights into Luffa cylindrica browning. *Biochemical and Biophysical Research Communications* **463**, 1243-1249 (2015).

37. Zhu, H. et al. De novo sequencing and analysis of the transcriptome during the browning of fresh-cut Luffa cylindrica 'Fusi-3' fruits. *PLOS ONE* **12**, e0187117 (2017).

38. Sun, J., Manmathan, H., Sun, C. & Peebles, C.A.M. Examining the transcriptional response of overexpressing anthranilate synthase in the hairy roots of an important medicinal plant Catharanthus roseus by RNA-seq. *BMC Plant Biology* **16** (2016).

39. Liu, L.D. et al. High-Throughput Transcriptome Analysis of the Leafy Flower Transition of Catharanthus roseus Induced by Peanut Witches’-Broom Phytoplasma Infection. *Plant and Cell Physiology* **55**, 942-957 (2014).

40. Kumar, S., Shah, N., Garg, V. & Bhatia, S. Large scale in-silico identification and characterization of simple sequence repeats (SSRs) from de novo assembled transcriptome of Catharanthus roseus (L.) G. Don. *Plant Cell Reports* **33**, 905-918 (2014).

41. Yang, C. et al. MicroRNA396-TargetedSHORT VEGETATIVE PHASE Is Required to Repress Flowering and Is Related to the Development of Abnormal Flower Symptoms by the Phyllody Symptoms1 Effector. *Plant Physiology* **168**, 1702-1716 (2015).

42. Verma, M., Ghangal, R., Sharma, R., Sinha, A.K. & Jain, M. Transcriptome analysis of Catharanthus roseus for gene discovery and expression profiling. *PLoS One* **9**, e103583 (2014).

43. Gongora-Castillo, E. et al. Development of transcriptomic resources for interrogating the biosynthesis of monoterpene indole alkaloids in medicinal plant species. *PLoS One* **7**, e52506 (2012).

44. Van Moerkercke, A. et al. The bHLH transcription factor BIS1 controls the iridoid branch of the monoterpenoid indole alkaloid pathway inCatharanthus roseus. *Proceedings of the National Academy of Sciences* **112**, 8130-8135 (2015).

45. Annadurai, R.S. et al. De Novo transcriptome assembly (NGS) of Curcuma longa L. rhizome reveals novel transcripts related to anticancer and antimalarial terpenoids. *PloS one* **8**, e56217 (2013).

46. Sahoo, A., Kar, B., Sahoo, S., Ray, A. & Nayak, S. Transcriptome profiling of Curcuma longa L. cv. Suvarna. *Genomics Data* **10**, 33-34 (2016).

47. Sahoo, A., Jena, S., Sahoo, S., Nayak, S. & Kar, B. Resequencing of Curcuma longa L. cv. Kedaram through transcriptome profiling reveals various novel transcripts. *Genomics Data* **9**, 160-161 (2016).

48. Hellinger, R. et al. Peptidomics of Circular Cysteine-Rich Plant Peptides: Analysis of the Diversity of Cyclotides fromViola tricolor by Transcriptome and Proteome Mining. *Journal of Proteome Research* **14**, 4851-4862 (2015).

49. Li, Y., Gou, J., Chen, F., Li, C. & Zhang, Y. Comparative Transcriptome Analysis Identifies Putative Genes Involved in the Biosynthesis of Xanthanolides in Xanthium strumarium L. *Frontiers in Plant Science* **7** (2016).

50. Huang, Q. et al. Differential Gene Expression between Leaf and Rhizome in Atractylodes lancea: A Comparative Transcriptome Analysis. *Frontiers in Plant Science* **7** (2016).

51. Ahmed, S. et al. The Transcript Profile of a Traditional Chinese Medicine, Atractylodes lancea, Revealing Its Sesquiterpenoid Biosynthesis of the Major Active Components. *PLOS ONE* **11**, e0151975 (2016).

52. Khan, S. et al. Comparative transcriptome analysis reveals candidate genes for the biosynthesis of natural insecticide in Tanacetum cinerariifolium. *BMC Genomics* **18** (2017).

53. Jiang, N.H. et al. Analysis of the transcriptome of Erigeron breviscapus uncovers putative scutellarin and chlorogenic acids biosynthetic genes and genetic markers. *PLoS One* **9**, e100357 (2014).

54. Zhang, W. et al. Transcriptomic comparison of the self-pollinated and cross-pollinated flowers of Erigeron breviscapus to analyze candidate self-incompatibility-associated genes. *BMC Plant Biology* **15** (2015).

55. Chen, R. et al. Deep Sequencing Reveals the Effect of MeJA on Scutellarin Biosynthesis in Erigeron breviscapus. *PLOS ONE* **10**, e0143881 (2015).

56. Lulin, H., Xiao, Y., Pei, S., Wen, T. & Shangqin, H. The first Illumina-based de novo transcriptome sequencing and analysis of safflower flowers. *PLoS One* **7**, e38653 (2012).

57. Liu, X. et al. De Novo Sequencing and Analysis of the Safflower Transcriptome to Discover Putative Genes Associated with Safflor Yellow in Carthamus tinctorius L. *International Journal of Molecular Sciences* **16**, 25657-25677 (2015).

58. Wang, H. et al. Next-generation sequencing of the Chrysanthemum nankingense (Asteraceae) transcriptome permits large-scale unigene assembly and SSR marker discovery. *PLoS One* **8**, e62293 (2013).

59. Ren, L. et al. A transcriptomic analysis of Chrysanthemum nankingense provides insights into the basis of low temperature tolerance. *BMC Genomics* **15**, 844 (2014).

60. Sun, J. et al. Identification of differentially expressed genes in Chrysanthemum nankingense (Asteraceae) under heat stress by RNA Seq. *Gene* **552**, 59-66 (2014).

61. Won, S.Y. et al. Comparative transcriptome analysis reveals whole-genome duplications and gene selection patterns in cultivated and wild Chrysanthemum species. *Plant Molecular Biology* **95**, 451-461 (2017).

62. Wang, W., Wang, Y., Zhang, Q., Qi, Y. & Guo, D. Global characterization of Artemisia annua glandular trichome transcriptome using 454 pyrosequencing. *BMC Genomics* **10**, 465 (2009).

63. Graham, I.A. et al. The Genetic Map of Artemisia annua L. Identifies Loci Affecting Yield of the Antimalarial Drug Artemisinin. *Science* **327**, 328-331 (2010).

64. Nair, P. et al. Differentially expressed genes during contrasting growth stages of Artemisia annua for artemisinin content. *PLoS One* **8**, e60375 (2013).

65. Garg, A., Agrawal, L., Misra, R.C., Sharma, S. & Ghosh, S. Andrographis paniculata transcriptome provides molecular insights into tissue-specific accumulation of medicinal diterpenes. *BMC Genomics* **16** (2015).

66. Cherukupalli, N., Divate, M., Mittapelli, S.R., Khareedu, V.R. & Vudem, D.R. De novo Assembly of Leaf Transcriptome in the Medicinal Plant Andrographis paniculata. *Frontiers in Plant Science* **7** (2016).

67. Huang, Y. et al. Stable Internal Reference Genes for Normalizing Real-Time Quantitative PCR in Baphicacanthus cusia under Hormonal Stimuli and UV Irradiation, and in Different Plant Organs. *Frontiers in Plant Science* **8** (2017).

68. Huang, Q. et al. The first insight into transcriptome profile of herbaceous plant Nervilia fordii based on RNA-seq. *Plant Omics* **8**, 493 (2015).

69. Li, Q., Ding, G., Li, B. & Guo, S. Transcriptome Analysis of Genes Involved in Dendrobine Biosynthesis in Dendrobium nobile Lindl. Infected with Mycorrhizal Fungus MF23 (Mycena sp.). *Scientific Reports* **7** (2017).

70. Guo, X. et al. Analysis of the Dendrobium officinale transcriptome reveals putative alkaloid biosynthetic genes and genetic markers. *Gene* **527**, 131-138 (2013).

71. He, L. et al. Hybrid Sequencing of Full-Length cDNA Transcripts of Stems and Leaves in Dendrobium officinale. *Genes* **8**, 257 (2017).

72. Xu, M. et al. Transcriptome sequencing and development of novel genic SSR markers for Dendrobium officinale. *Molecular Breeding* **37** (2017).

73. Shen, C. et al. Identification and analysis of genes associated with the synthesis of bioactive constituents in Dendrobium officinale using RNA-Seq. *Scientific Reports* **7** (2017).

74. An, H. et al. Whole-Transcriptome Selection and Evaluation of Internal Reference Genes for Expression Analysis in Protocorm Development of Dendrobium officinale Kimura et Migo. *PLOS ONE* **11**, e0163478 (2016).

75. Meng, Y. et al. A transcriptome-wide, organ-specific regulatory map of Dendrobium officinale, an important traditional Chinese orchid herb. *Scientific Reports* **6** (2016).

76. Tsai, C. et al. Comparative transcriptome analysis of Gastrodia elata (Orchidaceae) in response to fungus symbiosis to identify gastrodin biosynthesis-related genes. *BMC Genomics* **17** (2016).

77. Zeng, X. et al. Transcriptomic analyses reveal clathrin-mediated endocytosis involved in symbiotic seed germination of Gastrodia elata. *Botanical Studies* **58** (2017).

78. Sun, Y. et al. Pyrosequencing of the Camptotheca acuminata transcriptome reveals putative genes involved in camptothecin biosynthesis and transport. *BMC Genomics* **12**, 533 (2011).

79. Liu, H., Wu, W., Hou, K., Chen, J. & Zhao, Z. Transcriptome changes in Polygonum multiflorum Thunb. roots induced by methyl jasmonate. *Journal of Zhejiang University-SCIENCE B* **16**, 1027-1041 (2015).

80. Liu, H., Wu, W., Hou, K., Chen, J. & Zhao, Z. Deep sequencing reveals transcriptome re-programming of Polygonum multiflorum thunb. roots to the elicitation with methyl jasmonate. *Molecular Genetics and Genomics* **291**, 337-348 (2016).

81. Hao, D. et al. De novo characterization of the root transcriptome of a traditional Chinese medicinal plant Polygonum cuspidatum. *Science China Life Sciences* **55**, 452-466 (2012).

82. Chen, C. & Li, A. Transcriptome Analysis of Differentially Expressed Genes Involved in Proanthocyanidin Accumulation in the Rhizomes of Fagopyrum dibotrys and an Irradiation-Induced Mutant. *Frontiers in Physiology* **7** (2016).

83. Liu, Y. et al. Deep sequencing and transcriptome analyses to identify genes involved in secoiridoid biosynthesis in the Tibetan medicinal plant Swertia mussotii. *Scientific Reports* **7**, 43108 (2017).

84. Zhang, X., Allan, A., Li, C., Wang, Y. & Yao, Q. De Novo Assembly and Characterization of the Transcriptome of the Chinese Medicinal Herb, Gentiana rigescens. *International Journal of Molecular Sciences* **16**, 11550-11573 (2015).

85. Zheng, K. et al. Analysis of the transcriptome of Marsdenia tenacissima discovers putative polyoxypregnane glycoside biosynthetic genes and genetic markers. *Genomics* **104**, 186-193 (2014).

86. Okada, T. et al. Comparative analysis of transcriptomes in aerial stems and roots of Ephedra sinica based on high-throughput mRNA sequencing. *Genomics Data* **10**, 4-11 (2016).

87. Groves, R.A. et al. Transcriptome Profiling of Khat (Catha edulis) and Ephedra sinica Reveals Gene Candidates Potentially Involved in Amphetamine-Type Alkaloid Biosynthesis. *PLOS ONE* **10**, e0119701 (2015).

88. Zhan, C. et al. Comprehensive Analysis of the Triterpenoid Saponins Biosynthetic Pathway in Anemone flaccida by Transcriptome and Proteome Profiling. *Frontiers in Plant Science* **7** (2016).

89. Tsanakas, G.F., Manioudaki, M.E., Economou, A.S. & Kalaitzis, P. De novo transcriptome analysis of petal senescence in Gardenia jasminoides Ellis. *BMC Genomics* **15**, 554 (2014).

90. Zhao, D. et al. Ameliorative effects of melatonin on dark-induced leaf senescence in gardenia (Gardenia jasminoides Ellis): leaf morphology, anatomy, physiology and transcriptome. *Scientific Reports* **7** (2017).

91. Dai, H. et al. Transcript assembly and quantification by RNA-Seq reveals differentially expressed genes between soft-endocarp and hard-endocarp hawthorns. *PLoS One* **8**, e72910 (2013).

92. Xu, J. et al. Transcriptome Analysis and Ultrastructure Observation Reveal that Hawthorn Fruit Softening Is due to Cellulose/Hemicellulose Degradation. *Frontiers in Plant Science* **7** (2016).

93. Garzon-Martinez, G.A., Zhu, Z.I., Landsman, D., Barrero, L.S. & Marino-Ramirez, L. The Physalis peruviana leaf transcriptome: assembly, annotation and gene model prediction. *BMC Genomics* **13**, 151 (2012).

94. Wang, G. et al. De novo characterization of the Lycium chinense Mill. leaf transcriptome and analysis of candidate genes involved in carotenoid biosynthesis. *Gene* **555**, 458-463 (2015).

95. Khaldun, A.B.M., Huang, W., Liao, S., Lv, H. & Wang, Y. Identification of MicroRNAs and Target Genes in the Fruit and Shoot Tip of Lycium chinense: A Traditional Chinese Medicinal Plant. *PLOS ONE* **10**, e0116334 (2015).

96. Gong, L. et al. LbCML38 and LbRH52, two reference genes derived from RNA-Seq data suitable for assessing gene expression in Lycium barbarum L. *Scientific Reports* **6** (2016).

97. Chen, C. et al. Characterization of the Lycium barbarum fruit transcriptome and development of EST-SSR markers. *PLOS ONE* **12**, e0187738 (2017).

98. Yuan, Y. et al. Genetic variation and metabolic pathway intricacy govern the active compound content and quality of the Chinese medicinal plant Lonicera japonica thunb. *BMC Genomics* **13**, 195 (2012).

99. Rai, A. et al. De novo transcriptome assembly and characterization of nine tissues of Lonicera japonica to identify potential candidate genes involved in chlorogenic acid, luteolosides, and secoiridoid biosynthesis pathways. *Journal of Natural Medicines* **71**, 1-15 (2017).

100. Chen, Z. et al. Transcriptome Analysis Reveals the Mechanism Underlying the Production of a High Quantity of Chlorogenic Acid in Young Leaves of Lonicera macranthoides Hand.-Mazz. *PLOS ONE* **10**, e0137212 (2015).

101. Zhang, Z et al. The mechanical wound transcriptome of three-year-old Aquilaria sinensis. *Y* 1106-1110 (2012).

102. Xu, Y. et al. Identification of genes related to agarwood formation: transcriptome analysis of healthy and wounded tissues of Aquilaria sinensis. *BMC Genomics* **14**, 227 (2013).

103. Ye, W. et al. Transcriptome Sequencing of Chemically Induced Aquilaria sinensis to Identify Genes Related to Agarwood Formation. *PLOS ONE* **11**, e0155505 (2016).

104. Wei, L. et al. Transcriptome analysis of Houttuynia cordata Thunb. by Illumina paired-end RNA sequencing and SSR marker discovery. *PLoS One* **9**, e84105 (2014).

105. Sui, C. et al. Comparison of root transcriptomes and expressions of genes involved in main medicinal secondary metabolites from Bupleurum chinense and Bupleurum scorzonerifolium, the two Chinese official Radix bupleuri source species. *PHYSIOLOGIA PLANTARUM* **153**, 230-242 (2015).

106. Song, T. et al. Comparative transcriptome of rhizome and leaf in Ligusticum Chuanxiong. *Plant Systematics and Evolution* **301**, 2073-2085 (2015).

107. Yang, J. et al. Differentially expressed genes in heads and tails of Angelica sinensis diels: Focusing on ferulic acid metabolism. *Chin J Integr Med* **23**, 779-785 (2017).

108. Sangwan, R.S., Tripathi, S., Singh, J., Narnoliya, L.K. & Sangwan, N.S. De novo sequencing and assembly of Centella asiatica leaf transcriptome for mapping of structural, functional and regulatory genes with special reference to secondary metabolism. *Gene* **525**, 58-76 (2013).

109. Kim, O., Jin, M., Lee, D. & Jetter, R. Characterization of the Asiatic Acid Glucosyltransferase, UGT73AH1, Involved in Asiaticoside Biosynthesis in Centella asiatica (L.) Urban. *International Journal of Molecular Sciences* **18**, 2630 (2017).

110. Jiang, Q. et al. De novo transcriptome assembly, gene annotation, marker development, and miRNA potential target genes validation under abiotic stresses in Oenanthe javanica. *Molecular Genetics and Genomics* **290**, 671-683 (2015).

111. Van Bakel, H. et al. The draft genome and transcriptome of Cannabis sativa. *Genome biology* **12**, R102 (2011).

112. Zhang, C. et al. Transcriptomic analysis of cut tree peony with glucose supply using the RNA-Seq technique. *Plant Cell Rep* **33**, 111-29 (2014).

113. Shi, Q. et al. Transcriptomic Analysis of Paeonia delavayi Wild Population Flowers to Identify Differentially Expressed Genes Involved in Purple-Red and Yellow Petal Pigmentation. *PLOS ONE* **10**, e0135038 (2015).

114. Zhang, X., Zhao, L., Xu, Z. & Yu, X. Transcriptome sequencing of Paeonia suffruticosa ‘Shima Nishiki’ to identify differentially expressed genes mediating double-color formation. *Plant Physiology and Biochemistry* **123**, 114-124 (2018).

115. Xie, D. et al. Next generation sequencing and transcriptome analysis of root bark from Paeonia suffruticosa cv. Feng Dan. *China Journal of Chinese Materia Medica*, 2954-2961 (2017).

116. Zhang, L., Chen, J., Li, Q. & Chen, W. Transcriptome-wide analysis of basic helix-loop-helix transcription factors in Isatis indigotica and their methyl jasmonate responsive expression profiling. *Gene* **576**, 150-159 (2016).

117. Zhou, Y. et al. Transcriptomic Analysis Reveals Differential Gene Expressions for Cell Growth and Functional Secondary Metabolites in Induced Autotetraploid of Chinese Woad (Isatis indigotica Fort.). *PLOS ONE* **10**, e0116392 (2015).

118. Wang, Y. et al. De novo transcriptome sequencing of radish (Raphanus sativus L.) and analysis of major genes involved in glucosinolate metabolism. *BMC Genomics* **14**, 836 (2013).

119. Xu, L. et al. De novo sequencing of root transcriptome reveals complex cadmium-responsive regulatory networks in radish (Raphanus sativus L.). *Plant Science* **236**, 313-323 (2015).

120. Mitsui, Y. et al. The radish genome and comprehensive gene expression profile of tuberous root formation and development. *Scientific Reports* **5** (2015).

121. Nie, S. et al. De novo transcriptome analysis in radish (Raphanus sativus L.) and identification of critical genes involved in bolting and flowering. *BMC Genomics* **17** (2016).

122. Nie, S. et al. Transcriptomic Analysis Identifies Differentially Expressed Genes (DEGs) Associated with Bolting and Flowering in Radish (Raphanus sativus L.). *Frontiers in Plant Science* **7** (2016).

123. Zhang, L. et al. Transcriptome analysis of leaf tissue of Raphanus sativus by RNA sequencing. *PLoS One* **8**, e80350 (2013).

124. Xue, H. et al. De novo transcriptome assembly and quantification reveal differentially expressed genes between soft-seed and hard-seed pomegranate (Punica granatum L.). *PLOS ONE* **12**, e0178809 (2017).

125. Ono, N.N. et al. Exploring the Transcriptome Landscape of Pomegranate Fruit Peel for Natural Product Biosynthetic Gene and SSR Marker Discovery(F). *J Integr Plant Biol* **53**, 800-13 (2011).

126. Luo, H. et al. Comparison of 454-ESTs from Huperzia serrata and Phlegmariurus carinatus reveals putative genes involved in lycopodium alkaloid biosynthesis and developmental regulation. *BMC Plant Biol* **10**, 209 (2010).

127. Luo, H. et al. Comparison of 454-ESTs from Huperzia serrata and Phlegmariurus carinatus reveals putative genes involved in lycopodium alkaloid biosynthesis and developmental regulation. *BMC Plant Biol* **10**, 209 (2010).

128. Yang, M. et al. Global transcriptome analysis of Huperzia serrata and identification of critical genes involved in the biosynthesis of huperzine A. *BMC Genomics* **18** (2017).

129. Hua, Y. et al. Transcriptomic analysis of Pseudostellariae Radix from different fields using RNA-seq. *Gene* **588**, 7-18 (2016).

130. Li, J. et al. De Novo Sequencing and Assembly Analysis of the Pseudostellaria heterophylla Transcriptome. *PLOS ONE* **11**, e0164235 (2016).

131. He, M., Wang, Y., Hua, W., Zhang, Y. & Wang, Z. De novo sequencing of Hypericum perforatum transcriptome to identify potential genes involved in the biosynthesis of active metabolites. *PloS one* **7**, e42081 (2012).

132. Zhang, G. et al. De novo Sequencing and Transcriptome Analysis of Pinellia ternata Identify the Candidate Genes Involved in the Biosynthesis of Benzoic Acid and Ephedrine. *Frontiers in Plant Science* **7** (2016).

133. Gille, S. et al. Deep sequencing of voodoo lily (Amorphophallus konjac): an approach to identify relevant genes involved in the synthesis of the hemicellulose glucomannan. *Planta* **234**, 515-526 (2011).

134. Li, W. et al. De Novo Assembly and Characterization of Pericarp Transcriptome and Identification of Candidate Genes Mediating Fruit Cracking in Litchi chinensis Sonn. *International Journal of Molecular Sciences* **15**, 17667-17685 (2014).

135. Pathak, A.K. et al. Transcriptional changes during ovule development in two genotypes of litchi (Litchi chinensis Sonn.) with contrast in seed size. *Scientific Reports* **6** (2016).

136. Chen, S. et al. 454 EST analysis detects genes putatively involved in ginsenoside biosynthesis in Panax ginseng. *Plant Cell Reports* **30**, 1593-1601 (2011).

137. Li, C. et al. Transcriptome analysis reveals ginsenosides biosynthetic genes, microRNAs and simple sequence repeats in Panax ginseng C. A. Meyer. *BMC Genomics* **14**, 245 (2013).

138. Luo, H. et al. Analysis of the transcriptome of Panax notoginseng root uncovers putative triterpene saponin-biosynthetic genes and genetic markers. *BMC Genomics* **12**, S5 (2011).

139. Liu, M. et al. Transcriptome analysis of leaves, roots and flowers of Panax notoginseng identifies genes involved in ginsenoside and alkaloid biosynthesis. *BMC Genomics* **16** (2015).

140. Liu, Y., Mi, Y., Zhang, J., Li, Q. & Chen, L. Illumina-based transcriptomic profiling of Panax notoginseng in response to arsenic stress. *Botanical Studies* **57** (2016).

141. Sun, C. et al. De novo sequencing and analysis of the American ginseng root transcriptome using a GS FLX Titanium platform to discover putative genes involved in ginsenoside biosynthesis. **11**, 262 (2010).

142. Wang, J. et al. Transcriptome profiling shows gene regulation patterns in ginsenoside pathway in response to methyl jasmonate in Panax Quinquefolium adventitious root. *Scientific Reports* **6** (2016).

143. Zhang, S. et al. De novo characterization of Panax japonicus C. A. Mey transcriptome and genes related to triterpenoid saponin biosynthesis. *Biochemical and Biophysical Research Communications* **466**, 450-455 (2015).

144. Rai, A. et al. RNA-seq Transcriptome Analysis of Panax japonicus, and Its Comparison with Other Panax Species to Identify Potential Genes Involved in the Saponins Biosynthesis. *Frontiers in Plant Science* **7** (2016).

145. Qing-tian, Z., Shu-tian, F., Yi-ming, Y., Pei-lei, X. & Jun, A. De novo transcriptome assembly of Schisandra chinensis Turcz. (Baill.). *Genomics Data* **10**, 153-154 (2016).

146. Li, J. et al. Transcriptome Analysis to Identify the Putative Biosynthesis and Transport Genes Associated with the Medicinal Components of Achyranthes bidentata Bl. *Frontiers in Plant Science* **7** (2016).

147. Zeng, S. et al. Development of a EST dataset and characterization of EST-SSRs in a traditional Chinese medicinal plant, Epimedium sagittatum (Sieb. Et Zucc.) Maxim. **11**, 94 (2010).

148. Li, M.J. et al. Transcriptome/degradome-wide identification of R. glutinosa miRNAs and their targets: the role of miRNA activity in the replanting disease. *PLoS One* **8**, e68531 (2013).

149. Li, M. et al. Analysis of integrated multiple ‘omics’ datasets reveals the mechanisms of initiation and determination in the formation of tuberous roots inRehmannia glutinosa. *Journal of Experimental Botany* **66**, 5837-5851 (2015).

150. Sun, P. et al. Transcriptome Analysis Reveals Putative Genes Involved in Iridoid Biosynthesis in Rehmannia glutinosa. *International Journal of Molecular Sciences* **13**, 13748-13763 (2012).

151. Sun, P. et al. Dynamic transcriptional profiling provides insights into tuberous root development in Rehmannia glutinosa. *Frontiers in Plant Science* **6** (2015).

152. Wu, B. et al. Comprehensive transcriptome analysis reveals novel genes involved in cardiac glycoside biosynthesis and mlncRNAs associated with secondary metabolism and stress response in Digitalis purpurea. *BMC Genomics* **13**, 15 (2012).

153. He, B. et al. Transcriptome analysis of Ginkgo biloba kernels. *Frontiers in Plant Science* **6** (2015).

154. Lin, X. et al. Functional genomics of a living fossil tree, Ginkgo, based on next-generation sequencing technology. *Physiol Plant* **143**, 207-18 (2011).

155. Zeng, J. et al. Integration of transcriptome, proteome and metabolism data reveals the alkaloids biosynthesis in Macleaya cordata and Macleaya microcarpa. *PLoS One* **8**, e53409 (2013).

156. Zeng, J. et al. Integration of transcriptome, proteome and metabolism data reveals the alkaloids biosynthesis in Macleaya cordata and Macleaya microcarpa. *PLoS One* **8**, e53409 (2013).

157. Baba, S.A. et al. Comprehensive transcriptome analysis of Crocus sativus for discovery and expression of genes involved in apocarotenoid biosynthesis. *BMC Genomics* **16** (2015).

158. Tian, H. et al. Analysis ofPolygala tenuifolia Transcriptome and Description of Secondary Metabolite Biosynthetic Pathways by Illumina Sequencing. *International Journal of Genomics* **2015**, 1-11 (2015).

159. Niu, J. et al. Integrated transcriptome sequencing and dynamic analysis reveal carbon source partitioning between terpenoid and oil accumulation in developing Lindera glauca fruits. *Scientific Reports* **5**, 15017 (2015).


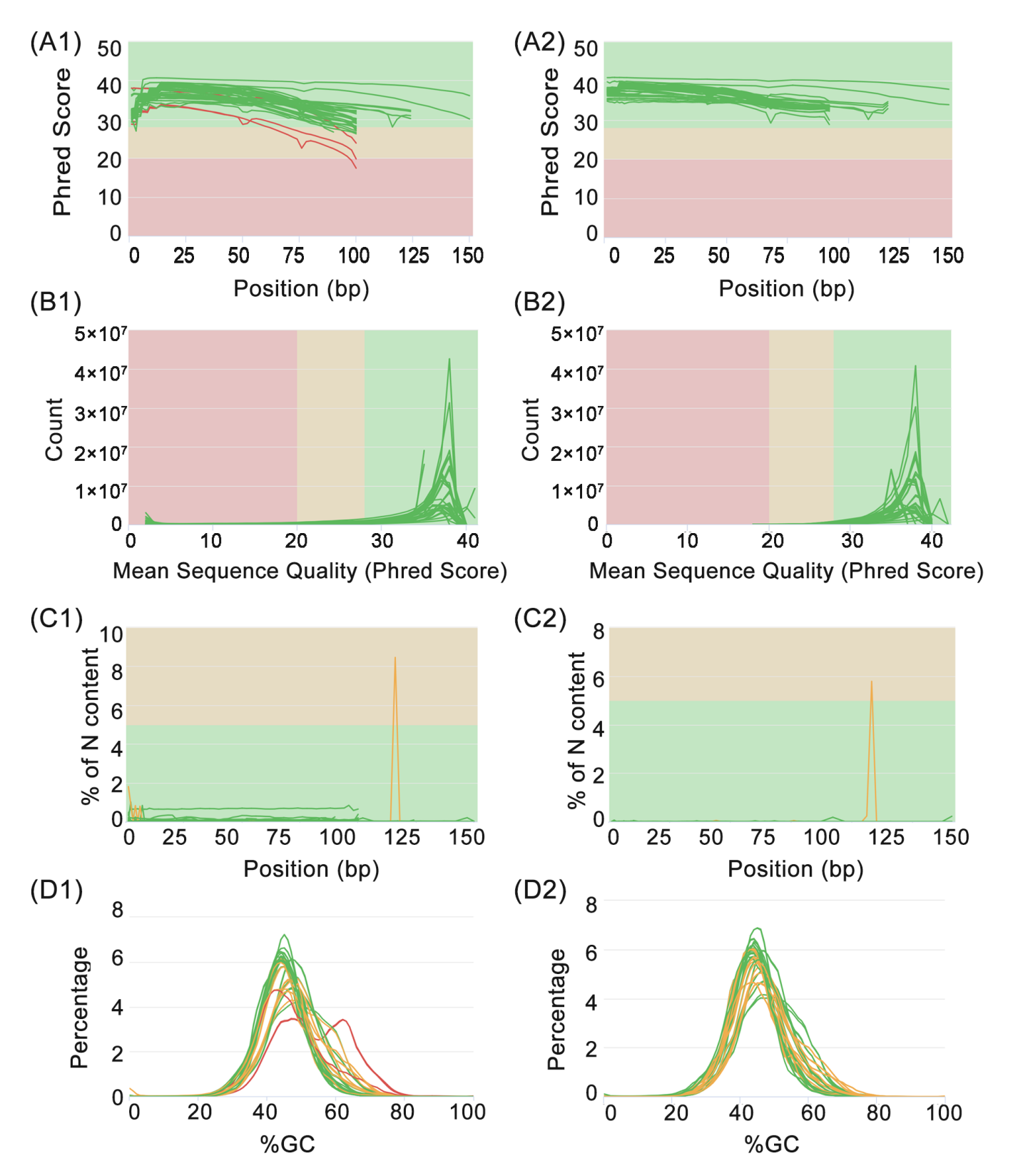


**Figure S1 FastQC results of each sample before and after quality control**

(A1, B1, C1, D1) Results of raw data. (A2, B2, C2, D2) Results of clean data. (A1-A2) Average quality scores by nt locations of reads. (B1-B2) Number distributions of reads in average quality scores. (C1-C2): Percentages of base N content by nt locations of reads. (D1-D2): Percentage distributions of sequence GC content, green for “pass”, yellow for “warn”, red for “fail”.


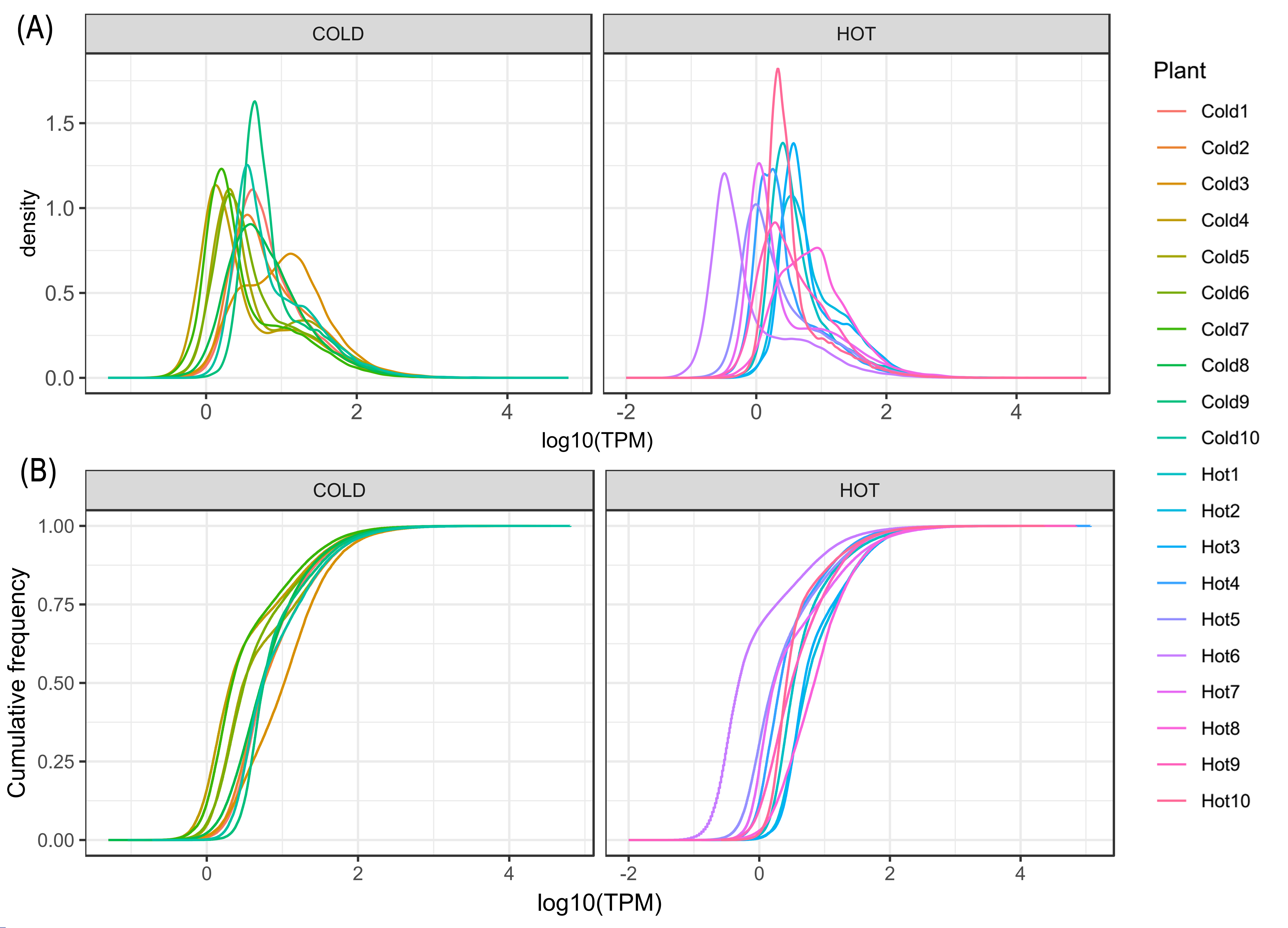


**Figure S3 Distribution of sample gene expression level.**

Gene expression levels were measure by log10(TPM). The different colors of the curves represented different species. (A) Density of RNA-seq gene expression level. (B) Cumulative frequency of RNA-seq gene expression level.

**Table S2. Classification and sampling sources of 20 plants and reference species**

| **Property** | **Sample id** | **Division** | **Class** | **Order** | **Family** | **Genus** | **Species** | **NCBI taxonomy id** |
| --- | --- | --- | --- | --- | --- | --- | --- | --- |
| Cold | cold1 | Magnoliophyta | Dicotyledoneae | Lamiales | Orobanchaceae | Rehmannia | *Rehmannia glutinosa* (Gaertn.) Libosch. | 99300 |
|  | cold2 | Magnoliophyta | Magnoliophyta | Lamiales | Lamiaceae | Dracocephalum | *Dracocephalum tanguticum* Maxim. | 1699510 |
|  | cold3 | Magnoliophyta | Magnoliophyta | Lamiales | Lamiaceae | Scutellaria | *Scutellaria baicalensis* Georgi | 65409 |
|  | cold4 | Magnoliophyta | Dicotyledoneae | Gentianales | Apocynaceae | Catharanthus | *Catharanthus roseus* (L.) G. Don | 4058 |
|  | cold5 | Magnoliophyta | Magnoliophyta | Lamiales | Acanthaceae | Andrographis | *Andrographis paniculata* (Burm. f.) Nees | 175694 |
|  | cold6 | Magnoliophyta | Dicotyledoneae | Gentianales | Gentianaceae | Swertia | *Swertia mussotii* Franch. | 137888 |
|  | cold7 | Magnoliophyta | Dicotyledoneae | Gentianales | Gentianaceae | Gentiana | *Gentiana rigescens* Franch. ex Hemsl. | 553056 |
|  | cold8 | Magnoliophyta | Dicotyledoneae | Gentianales | Rubiaceae | Gardenia | *Gardenia jasminoides* Ellis | 114476 |
|  | cold9 | Magnoliophyta | Dicotyledoneae | Dipsacales | Caprifoliaceae | Lonicera | *Lonicera japonica* Thunb. | 105884 |
|  | cold10 | Magnoliophyta | Dicotyledoneae | Brassicales | Brassicaceae | Isatis | *Isatis tinctoria* Fort. | 161756 |
| Hot | hot1 | Magnoliophyta | Liliopsida | Asparagales | Amaryllidaceae | Allium | *Allium fistulosum* L. | 35875 |
|  | hot2 | Magnoliophyta | Dicotyledoneae | Lamiales | Lamiaceae | Isodon | *Isodon rubescens* (Hemsl.) Hara | 587669 |
|  | hot3 | Magnoliophyta | Liliopsida | Zingiberales | Zingiberaceae | Curcuma | *Curcuma longa* L. | 136217 |
|  | hot4 | Magnoliophyta | Dicotyledoneae | Asterales | Asteraceae | Atractylodes | *Atractylodes lancea* (Thunb.) DC. | 41486 |
|  | hot5 | Magnoliophyta | Dicotyledoneae | Asterales | Asteraceae | Erigeron | *Erigeron breviscapus* (Vant.) Hand. -Mazz. | 244311 |
|  | hot6 | Gnetophyta | Gnetopsida | Ephedrales | Ephedraceae | Ephedra | *Ephedra sinica* Stapf | 33152 |
|  | hot7 | Magnoliophyta | Dicotyledoneae | Ranunculales | Ranunculaceae | Anemone | *Anemone flaccida*Fr. Shmidt | 174164 |
|  | hot8 | Magnoliophyta | Liliopsida | Alismatales | Araceae | Pinellia | *Pinellia ternata* (Thunb.) Berit | 199225 |
|  | hot9 | Magnoliophyta | Dicotyledoneae | Apiales | Araliaceae | Panax | *Panax notoginseng* (Burk.) F.H. Chen ex C.Chow | 44586 |
|  | hot10 | Magnoliophyta | Dicotyledoneae | Laurales | Lauraceae | Lindera | *Lindera glauca* (Sieb. et Zucc.) Bl | 332435 |
| / | Reference | Magnoliophyta | Dicotyledoneae | Brassicales | Brassicaceae | Arabidopsis | *Arabidopsis thaliana* | 3702 |

**Table S3. Sample sequencing data quality evaluation**

| **Property** | **Sample id** | **Sequencing platform** | **Raw reads** | | | | | | **Clean reads** | | | | |
| --- | --- | --- | --- | --- | --- | --- | --- | --- | --- | --- | --- | --- | --- |
|  |  |  | **Read num.** | **GC(%)** | **Q20** | **Q30** | **Length(bp)** | **Size(Gb)** | **Read num.** | **GC%** | **Q20** | **Q30** | **Length(bp)** |
| Cold | cold1 | Illumina HiSeq 2000 | 20316667 | 45 | 95.72% | 81.89% | 90 | 3.7 | 19200516 | 44 | 100.00% | 91.95% | 79 |
|  | cold2 | Illumina HiSeq 4000 | 21679517 | 48 | 84.04% | 77.05% | 151 | 6.5 | 13593496 | 48 | 100.00% | 97.56% | 136 |
|  | cold3 | Illumina HiSeq 2000 | 22817910 | 45 | 86.83% | 74.67% | 100 | 4.6 | 21680378 | 45 | 100.00% | 90.35% | 89 |
|  | cold4 | Illumina HiSeq 2000 | 57546161 | 44 | 92.20% | 86.74% | 100 | 11.5 | 57049162 | 44 | 100.00% | 96.61% | 91 |
|  | cold5 | Illumina HiSeq 2000 | 50892666 | 48 | 95.35% | 85.51% | 101 | 10.3 | 46667500 | 47 | 100.00% | 94.32% | 90 |
|  | cold6 | Illumina HiSeq 2000 | 46991756 | 43 | 96.72% | 88.75% | 101 | 9.5 | 43079023 | 43 | 100.00% | 96.23% | 91 |
|  | cold7 | Illumina HiSeq 2000 | 53933882 | 43 | 96.75% | 86.85% | 100 | 10.8 | 48553796 | 42 | 100.00% | 95.09% | 90 |
|  | cold8 | Illumina HiSeq 2000 | 25167836 | 45 | 99.77% | 88.83% | 90 | 4.5 | 24543475 | 45 | 100.00% | 94.21% | 80 |
|  | cold9 | Illumina HiSeq 2000 | 13493109 | 43 | 97.12% | 86.71% | 101 | 2.7 | 12333697 | 43 | 100.00% | 94.64% | 90 |
|  | cold10 | Illumina HiSeq 2000 | 16989722 | 47 | 93.07% | 85.12% | 100 | 3.4 | 15966731 | 47 | 100.00% | 95.16% | 91 |
| Hot | hot1 | Illumina HiSeq 2000 | 24894222 | 43 | 88.33% | 75.31% | 101 | 5 | 20333238 | 43 | 100.00% | 91.68% | 90 |
|  | hot2 | Illumina HiSeq 2000 | 16732738 | 48 | 99.00% | 91.01% | 125 | 4.2 | 14896528 | 48 | 100.00% | 94.75% | 114 |
|  | hot3 | Illumina NextSeq 500 | 25797778 | 46 | 98.68% | 93.24% | 76 | 3.9 | 24637210 | 46 | 100.00% | 96.59% | 68 |
|  | hot4 | Illumina HiSeq 2000 | 24441642 | 44 | 92.17% | 80.40% | 125 | 6.1 | 21508787 | 44 | 100.00% | 89.97% | 113 |
|  | hot5 | Illumina HiSeq 2000 | 68680144 | 44 | 89.41% | 80.61% | 101 | 13.9 | 59988628 | 43 | 100.00% | 93.79% | 91 |
|  | hot6 | Illumina HiSeq 2000 | 95676077 | 49 | 95.38% | 84.73% | 100 | 19.1 | 87043718 | 48 | 100.00% | 97.21% | 88 |
|  | hot7 | Illumina HiSeq 2000 | 51106163 | 45 | 94.28% | 90.14% | 90 | 9.2 | 50656572 | 44 | 100.00% | 96.74% | 82 |
|  | hot8 | Illumina HiSeq 2000 | 33846493 | 53 | 97.12% | 82.30% | 100 | 6.8 | 18903199 | 46 | 100.00% | 93.20% | 90 |
|  | hot9 | Illumina HiSeq 2000 | 32629487 | 44 | 99.79% | 90.97% | 90 | 5.9 | 31857050 | 43 | 100.00% | 94.62% | 81 |
|  | hot10 | Illumina HiSeq 2000 | 30854654 | 48 | 80.68% | 61.03% | 100 | 6.2 | 23319530 | 47 | 100.00% | 86.43% | 87 |

**Table S4 Statistics for novo transcriptome assembly of each sample**

| **Property** | **Sample id** | **Unigene Num.** | **Total base** | **Largest len** | **Average len** | **Median len** | **N50** | **GC(%)** | **BUSCO(%)** | **Trasrate Scroe** | **Trasrate Mapped Ratio(%)** |
| --- | --- | --- | --- | --- | --- | --- | --- | --- | --- | --- | --- |
| Cold | cold1 | 49202 | 34384293 | 7996 | 698.8 | 403 | 1137 | 41.81 | 56.5 | 0.38047 | 88.80 |
|  | cold2 | 39796 | 37879320 | 8874 | 951.8 | 583 | 1610 | 43.3 | 73.7 | 0.29807 | 89.29 |
|  | cold3 | 38010 | 35592509 | 8803 | 936.4 | 618 | 1531 | 42.33 | 66.9 | 0.17044 | 86.89 |
|  | cold4 | 46139 | 43688593 | 14744 | 946.9 | 455 | 1837 | 39.79 | 67.8 | 0.4721 | 86.73 |
|  | cold5 | 43886 | 48323024 | 20312 | 1101.1 | 513 | 2155 | 42.29 | 71 | 0.45434 | 90.59 |
|  | cold6 | 57194 | 47240759 | 12803 | 826 | 437 | 1489 | 39.81 | 70.3 | 0.42544 | 89.34 |
|  | cold7 | 74395 | 56540577 | 15654 | 760 | 385 | 1396 | 40.17 | 67.7 | 0.39402 | 86.69 |
|  | cold8 | 49912 | 32411262 | 7371 | 649.4 | 412 | 944 | 42.1 | 44 | 0.3654 | 89.09 |
|  | cold9 | 50771 | 43230373 | 15707 | 851.5 | 419 | 1597 | 40.63 | 75.4 | 0.53457 | 89.95 |
|  | cold10 | 33605 | 33697376 | 14840 | 1002.7 | 614 | 1683 | 43.51 | 75.6 | 0.51625 | 89.72 |
| Hot | hot1 | 60945 | 44407875 | 15201 | 728.7 | 403 | 1214 | 39.21 | 72.8 | 0.43668 | 89.70 |
|  | hot2 | 39191 | 38066622 | 13566 | 971.3 | 544 | 1703 | 42.88 | 71.1 | 0.4436 | 89.16 |
|  | hot3 | 46114 | 37856015 | 15596 | 820.9 | 430 | 1469 | 44.29 | 64.8 | 0.4039 | 87.00 |
|  | hot4 | 70043 | 51351000 | 12322 | 733.1 | 401 | 1261 | 41.96 | 67.2 | 0.33111 | 84.30 |
|  | hot5 | 78370 | 62703689 | 13317 | 800.1 | 462 | 1319 | 38.8 | 73.8 | 0.3431 | 86.56 |
|  | hot6 | 102400 | 59092623 | 10771 | 577.1 | 354 | 796 | 41.27 | 56.4 | 0.27428 | 84.52 |
|  | hot7 | 40422 | 36424301 | 15874 | 901.1 | 515 | 1540 | 42.24 | 77.3 | 0.52629 | 90.73 |
|  | hot8 | 51002 | 32345986 | 8863 | 634.2 | 417 | 896 | 45.11 | 51.4 | 0.09014 | 78.07 |
|  | hot9 | 68228 | 41340975 | 7425 | 605.9 | 402 | 834 | 41.13 | 50.2 | 0.35169 | 87.53 |
|  | hot10 | 65885 | 45022922 | 12944 | 683.4 | 355 | 1205 | 43.59 | 71.1 | 0.46261 | 86.42 |

BUSCO(%), the percentage of complete genes found in each species.

Trasrate Mapped Ratio(%), the percentage of clean reads mapping to the assembled contigs.


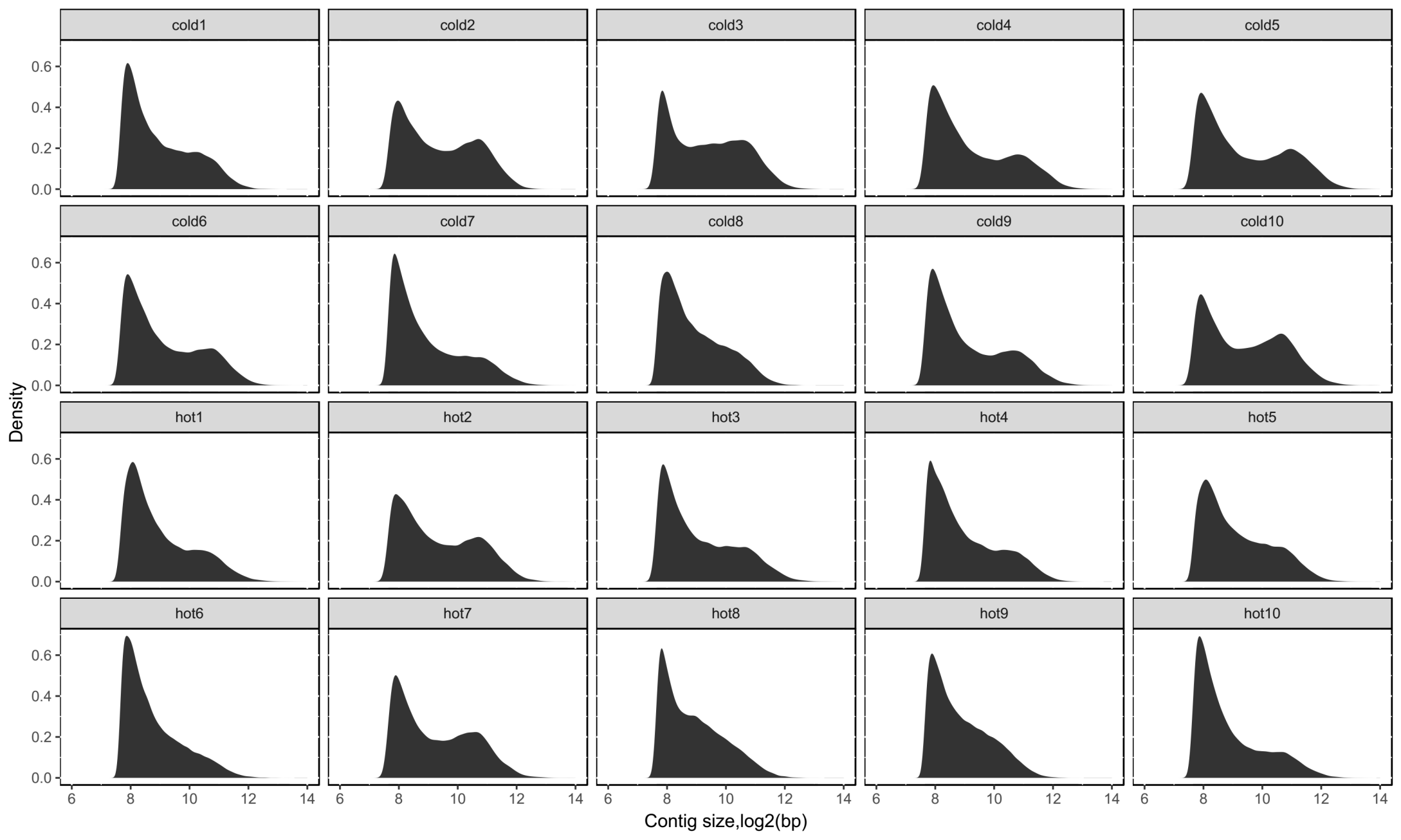


**Figure S2 The length distribution of unigenes.**
